# EPS8 phosphorylation by Src modulates its oncogenic functions

**DOI:** 10.1038/s41416-020-0976-6

**Published:** 2020-07-09

**Authors:** Linah A. Shahoumi, Hesam Khodadadi, Husam Bensreti, Babak Baban, W. Andrew Yeudall

**Affiliations:** 1 Department of Oral Biology and Diagnostic Sciences, Augusta University, Augusta, GA USA; 2 The Graduate School, Augusta University, Augusta, GA USA; 3 Georgia Cancer Center, Augusta University, Augusta, GA USA

**Keywords:** Oral cancer, Kinases

## Abstract

**Background:**

EPS8 is a scaffolding protein that regulates proliferation, actin dynamics and receptor trafficking. Its expression is increased in cancer, enhancing mitogenesis, migration and tumorigenesis. Src phosphorylates EPS8 at four tyrosine residues, although the function is unknown. Here we investigated the pro-oncogenic role of EPS8 tyrosine phosphorylation at Src target sites in HNSCC.

**Methods:**

Plasmids expressing EPS8 Src-mediated phosphorylation site mutants (Y485F, Y525F, Y602F, Y774F and all four combined [FFFF]) were expressed in cells containing a normal endogenous level of EPS8. In addition, cells were treated with dasatinib to inhibit Src activity. EPS8 downstream targets were evaluated by western blotting. Wound closure, proliferation, immunofluorescence and tumorgenicity assays were used to investigate the impact of phenylalanine mutations on EPS8 biological functions.

**Results:**

FOXM1, AURKA, and AURKB were decreased in cells expressing FFFF- and Y602F-EPS8 mutants, while cells harbouring the Y485F-, Y525F- and Y774F-EPS8 mutants showed no differences compared to controls. Consistent with this, dasatinib decreased the expression of EPS8 targets. Moreover, Y602F- and FFFF-EPS8 mutants reduced mitogenesis and motility. Strikingly though, FFFF- or Y602F-EPS8 mutants actually promoted tumorigenicity compared with control cells.

**Conclusions:**

Phosphorylation of EPS8 at Y602 is crucial for signalling to the cell cycle and may provide insight to explain reduced efficacy of dasatinib treatment.

## Background

Squamous cell carcinoma of the head and neck (HNSCC) is a common malignancy with high morbidity and mortality rates, affecting 630,000 patients worldwide with 50,000 new cases in the United States annually.^1,2^ The treatment options for HNSCC include surgery, radiotherapy, chemotherapy and, more recently, the use of targeted therapeutic agents.^3–5^ In spite of these advanced treatment options, the prognosis remains unsatisfactory. Five-year overall survival rates are dependent on the stage of the tumour with 85.2% survival with stage I disease, 82.9% stage II, 56.3% stage III and 42.6% stage IV,^6^ and the median overall survival for recurrent or metastatic (RM) HNSCC remains <1 year.^4,7^ There are multiple key genetic components promoting HNSCC development, one of which is overexpression or constitutive activation of the epidermal growth factor receptor (EGFR).^8–10^ EGFR is a receptor tyrosine kinase that has been found to be overexpressed in 80–100% of advanced-stage HNSCC.^11,12^ This kinase transduces signals through multiple pathways to promote cell proliferation, motility and survival.^13–18^ EGFR pathway substrate 8 (EPS8) is one of the mediators involved in post-receptor EGFR signalling.^19,20^ EPS8 is a scaffolding protein that exists as a dimer^21^ and shows several structural features: a Src homology 3 (SH3) domain, a split pleckstrin homology (PH) domain, several proline-rich regions, an EGFR-binding region, and a C-terminal effector region.^22^ The SH3 domain of EPS8 is reported to interact with RN-tre (USP6NL),^23^ Shc,^24^ Shb^25^ and Abi1,^26^ regulating cell proliferation, actin dynamics and receptor trafficking.^27,28^ EPS8 also binds to the juxtamembrane region of the EGFR^19^ and also to Sos-1^29^ and Src.^30,31^ EPS8 expression is increased in a range of human cancers, including HNSCC.^32^ Previous studies in our laboratory have shown that overexpression of EPS8 promotes cell proliferation and migration in part by inducing expression of the FOXM1 transcription factor, CXC-chemokine expression and matrix metalloproteinase-9 activity and expression, mediated by phosphoinositide 3-kinase (PI3K)/Akt-dependent mechanisms.^32,33^ Furthermore, studies showed that twofold overexpression of EPS8 is sufficient to convert non-tumorigenic cells to a tumorigenic phenotype in an orthotopic transplantation assay.^32^ The non-receptor tyrosine kinase Src modulates signalling downstream of multiple receptors including platelet-derived growth factor receptor and EGFR family receptors, regulating cell proliferation, migration and apoptosis.^34^ Src activity is closely correlated with solid tumour progression, metastasis and poor prognosis.^35–37^ Previous studies have demonstrated EPS8 overexpression in v-Src-transformed cells.^31,38^ Moreover, EPS8 was identified as a phosphorylation substrate for Src, and it is reported to phosphorylate EPS8 at four tyrosine residues: Y485, Y525, Y602, and Y774.^30^ To date, the impact of phosphorylation on biochemical functions of EPS8 is still unexplored, although it is likely that Src activity directed at EPS8 will be crucial for tumour progression. Deciphering how phosphorylation at the putative Src target sites in EPS8 affects its biochemical functions will likely provide insight into the key pathways that are deregulated in HNSCC. Therefore, in the current study, we have investigated biochemical and biological consequences of Src-mediated EPS8 phosphorylation in HNSCC.

## Methods

### Cell lines and culture conditions

HN4 cells, derived from a human papillomavirus-negative primary tongue tumour, express low endogenous levels of EPS8 similar to normal keratinocytes, whereas HN12 cells, derived from a synchronous lymph node metastasis in the same patient, express high endogenous levels of EPS8.^39^ Cell lines were obtained from Dr. J. F. Ensley (Wayne State University). Cells overexpressing unmodified EPS8 or its phenylalanine mutants were selected in the presence of 1 μg/ml puromycin or 400 μg/ml G418, respectively, in Dulbecco's modified Eagle's medium containing 10% cosmic calf serum, at 37 °C in a humidified atmosphere of 90% air/10% CO_2_. Prior to and during experimental assays, all cells were cultured in antibiotic-free media.

### Generation of mutations, plasmids and transfections

Expression plasmids encoding EPS8 with amino acid substitutions to phenylalanine (F) instead of tyrosine were created at the four putative Src phosphorylation sites as single mutants (Y485F, Y525F, Y602F and Y774F), all four combined (FFFF) and all sites except Y602 (FFF). All mutations were prepared using the QuikChange Site-directed Mutagenesis Kit (Agilent Technologies, Santa Clara, CA) following the instructions provided by the manufacturer. A plasmid containing the human EPS8 cDNA sequence, engineered to express an amino-terminal YFP tag (Genecopoeia; Rockville, MD), was used as template. All plasmids were sequence-verified before use. HN4 cells were nucleofected using a 4D-Nucleofector X unit (Lonza, Basel, Switzerland) with 1 μg of plasmid DNA. After 48 h, G418 was added to a final concentration of 400 μg/ml, and G418-resistant cells were selected and screened for the expression of EPS8 mutants.

### Cell treatment, lysis and western blotting

HN4 cells were treated with 400 nM dasatinib (Millipore-Sigma, St. Louis, MO) or the vehicle, dimethyl sulfoxide (DMSO; Fisher Scientific, Hampton, NH) for 24 h. Cells were washed twice in ice-cold phosphate-buffered saline (PBS), and whole-cell lysates were prepared by scraping cells into ice-cold modified RIPA lysis buffer [50 mM Tris–HCl (pH 7.4), 150 mM NaCl, 1% Triton, 0.5% deoxycholate] containing 1 mM phenylmethylsulfonyl fluoride, 10 μg/ml aprotinin, 10 μg/ml leupeptin and 1 mM sodium orthovanadate. In some experiments, cells were pretreated with 100 μM pervanadate for 10 min prior to lysis. The lysates were cleared by centrifugation, and the supernatants were collected. Analysis of protein expression was carried out as described previously.^40^ Blots were blocked in 5% skimmed milk in Tween-Tris buffered saline (TTBS) [10 mM Tris–HCl (pH 7.6), 0.5% Tween 20, 150 mM NaCl] for 1 h at room temperature. Western blot analysis was carried out with multiple primary antibodies as follows: EPS8 (610144) and AKT (610860) were from BD Biosciences (San Jose, CA); vimentin (V6389) was from Millipore Sigma (St. Louis, MO); FOXM1 (sc-502), pERK (sc-7383), phosphatase and tensin homologue (PTEN; sc-7974) and extracellular signal-regulated kinase 2 (ERK2; sc-154) were from Santa Cruz Biotechnology (Santa Cruz, CA); Aurora A kinase (3092), Aurora B kinase (3094), Src (2123), Src-pY416 (6943), phospho-AKT (Ser 473; 4060), phosphotyrosine pTyr-1000 (8954) and glyceraldehyde 3-phosphate dehydrogenase (2118) were all from Cell Signaling Technology (Danvers, MA). Following primary antibody incubation, the blots were washed 3× in TTBS for 10 min each, incubated with horseradish peroxidase (HRP)-conjugated secondary antibodies (goat anti-rabbit immunoglobulin G (IgG)-HRP-linked antibody (7074) or horse anti-mouse IgG-HRP-linked antibody (7076), both from Cell Signaling Technology, for 1 h at room temperature, washed 3× in TTBS and the specific signals were detected with an enhanced chemiluminescence substrate (PerkinElmer, Waltham, MA) followed by exposure to X-ray film or digital imaging (C-Digit; Li-Cor, Lincoln, NE).

For immunoprecipitation experiments, cell lysates were precleared by incubation with protein A/G agarose (Pierce, Rockford, IL) at 4 °C for 1 h. Subsequently, 1 mg aliquots of total cellular proteins were incubated with 1 μg of primary antibody or normal IgG as control, overnight at 4 °C with rotation. Immune complexes were captured using protein A/G agarose, washed 6× in cold lysis buffer and then resuspended in 1× sample buffer and heated to 95 °C for 10 min prior to electrophoresis and western blotting as described above.

### Cell proliferation assay

A total of 1 × 10^4^ cells were seeded in quadruplicate in 12-well culture plates. Cells were trypsinised and counted using a haemocytometer on each of the 7 consecutive days. For 3-(4,5-dimethylthiazol-2-yl)-2,5-diphenyltetrazolium bromide (MTT) assays, cells were plated in 12-well plates in two groups. At day 1, 0.1 vol. of MTT reagent (ThermoFisher, Waltham, MA) was added to each well for the first group. After 4 h incubation at 37 °C, the medium was aspirated, 1 ml of solubilisation buffer (10% sodium dodecyl sulfate in 0.01 M HCl) was added for 4 h and the absorbance was measured at 570 nm in a microtiter plate reader. The second group was cultured for 7 days and processed as before. Relative growth was calculated by subtracting the absorbance at day 1 from the absorbance at day 7.

### EdU (5-ethynyl-2’-deoxyuridine) incorporation assay

DNA synthesis in asynchronous cell populations was measured by incorporation of EdU, using a commercially available kit (Click-It EdU; Invitrogen Life Technologies, Carlsbad, CA) according to the manufacturer’s protocol. Briefly, cells were plated in triplicate on coverslips, pulsed with 10 μM EdU for 30 min at 37 °C, washed, labelled with Alexa Fluor 594, counterstained with 4,6-diamidino-2-phenylindole (DAPI), mounted and imaged using a fluorescent microscope (Keyence Corporation, Itasca, IL). The percentage of EdU-positive cells (EdU labelling index) was calculated by counting the number of Alexa Fluor 594-stained cells in two ×200 fields for each of three triplicate coverslips and dividing by the total cell number per field, expressed as a percentage. Experiments were repeated twice, and data were pooled for statistical analysis.

### Flow cytometry

Analysis of DNA content was carried out essentially as described before.^41^ Cells were cultured to 70% confluence, trypsinised, washed in PBS and fixed in 70% ethanol with vortexing. Cells were pelleted by centrifugation, resuspended in PBS, stained with 50 μg/ml propidium iodide and then analysed on a flow cytometer (FACSCalibur, BD Biosciences, San Jose, CA).

### Migration assay

To measure migration, 2 × 10^4^ cells were plated in triplicate in culture inserts (Ibidi, Fitchburg, WI) in 12-well cell culture plates and incubated at 37 °C overnight. On the following day, the inserts were removed and the cell-free gap was measured immediately at three specific points using a light microscope under a ×10 objective. After 7 h incubation at 37 °C, the cell-free gap was measured at the same three points. The migration rate was calculated by subtracting the width of the gap at 7 h from the starting width and dividing by the time.

### Immunofluorescence

Cells were grown on glass coverslips, fixed in cold methanol for 20 min at room temperature, permeabilised in 0.1% Triton X-100 (Fisher Scientific, Hampton, NH), blocked with 3% bovine serum albumin (BSA) in TTBS for 1 h at room temperature and then incubated with either anti-vimentin (V6389, Millipore Sigma, St. Louis, MO) diluted 1:250 in 3% BSA/TTBS or anti-pan-keratin (MA5–13156, ThermoFisher, Waltham, MA) diluted 1:250 in 3% BSA/TTBS overnight at 4 °C. As a negative control, samples were incubated with the equivalent concentration of normal mouse IgG. The coverslips were washed 3× in TTBS and incubated with goat anti-mouse IgG Alexa Fluor 488-conjugated secondary antibody (ThermoFisher, Waltham, MA) diluted 1:500 with 3% BSA/TTBS in the dark for 1 h at room temperature. The samples were subsequently stained with DAPI for 20 min and washed, and then coverslips were mounted using Vectashield (Vector Labs, Burlingame, CA) and visualised by fluorescence microscopy. As a negative control, coverslips were processed with normal mouse IgG at the equivalent concentration as the antibodies used.

### Orthotopic tumorigenicity assay

All animal experiments were carried out with approval of the Augusta University Institutional Animal Care and Use Committee (protocol number 2015–0736). Cells were trypsinised, counted and resuspended in serum-free medium. In all, 1 × 10^5^ cells in a total volume of 50 μl were injected into the tongues of 7-week-old nu/nu athymic mice (Charles River Laboratories, Wilmington, MA), previously anaesthetised with a cocktail of ketamine and xylazine (50–100 mg/kg ketamine; 2–11 mg/kg xylazine by the standard intraperitoneal route, to enable sufficient depth of anaesthesia as well as rapid induction and recovery). Four mice were used per group, based on similar previous experiments. As a positive control, 5 × 10^4^ HN12 cells were injected into the tongue. All experiments were performed inside a Class II biosafety cabinet during the animal’s light time cycle. Animals were monitored visually in their home cages to ensure recovery from anaesthesia and then monitored daily for tumour formation until they were euthanised (CO_2_ inhalation and bilateral thoracotomy) after becoming premorbid. Tumours were excised from euthanised animals and tumour mass was determined for all groups using the formula V = (*W*^2^ × *L*)/2, where *V* is the tumour volume, *W* is the tumour width and *L* is the tumour length. Tumour tissues were sectioned, fixed, stained with haematoxylin and eosin and examined under a light microscope.

### Immunohistochemistry

Detection of EPS8 in mouse tumour sections was carried out as follows. Five-micrometre tissue sections were dewaxed in Safe-Clear (Fisher Scientific, Pittsburgh, PA) and rehydrated, and antigen retrieval was performed by incubation in Retrievagen A (BD Biosciences, CA) at 95 °C for 30 min. After cooling to ambient temperature, endogenous peroxidase was blocked by incubation in 3% hydrogen peroxide for 15 min; slides were washed in TBS pH 8.0 and then blocked using a commercially available kit (M.O.M. Immunodetection Kit—Peroxidase; Vector Laboratories, Burlingame, CA). Sections were then incubated with EPS8 antibody (12.5 μg/ml) or the equivalent concentration of normal mouse IgG as control at ambient temperature for 1 h, washed in TBS and then incubated sequentially with biotinylated secondary antibody and streptavidin peroxidase reagent, developed using 3, 3’-diaminobenzidine substrate, counterstained with haematoxylin, dehydrated, mounted and imaged by brightfield microscopy (Keyence Corporation, Itasca, IL).

### RNA sequencing

For whole-transcriptome analysis, cells were cultured to 70% confluence under standard culture conditions. Alternatively, HN4 cells were treated with 400 nM dasatinib or equivalent volume of the vehicle (DMSO) as a control for 24 h. Triplicate cultures were lysed individually in Trizol, total RNA was prepared and then library construction, sequencing and bioinformatics analyses were carried out by a commercial source (Novogene, Sacramento, CA).

### Statistical analysis

Student’s *t* test or one-way analysis of variance with post multiple comparisons were performed using the GraphPad Prism software to analyse the data obtained from western blotting, migration, proliferation, immunostaining and tumorigenicity experiments. *p* values <0.05 were considered to be statistically significant.

## Results

### Blocking tyrosine phosphorylation at the Src target sites in EPS8 reduces the expression of cell cycle regulators

**Fig. 1:**
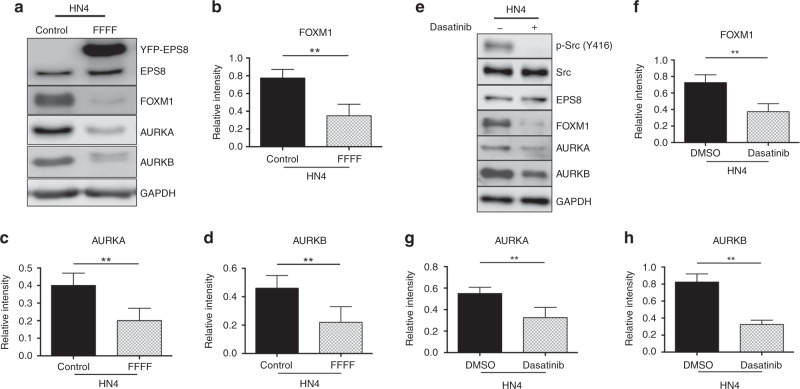
Effect of blocking Src-mediated EPS8 phosphorylation on EPS8 downstream targets. **a** HN4 cells were stably transfected with an EPS8 expression plasmid encoding the EPS8-FFFF mutant. Total cell lysates were prepared, resolved by SDS-PAGE and western blotted with the indicated antibodies. **b**–**d** Relative expression of FOXM1, AURKA and AURKB, normalised to GAPDH expression, is shown. Data are representative of independent biological experiments performed at least three times. **p < 0.01. **e** HN4 cells were incubated in the presence of dasatinib (400 nM) or an equivalent volume of DMSO. Twenty-four hours later, total cell lysates were prepared, resolved by SDS-PAGE and western blotted with the indicated antibodies. **f**–**h**. Relative expression of total and Y416-phosphorylated Src, FOXM1, AURKA and AURKB, normalised to GAPDH expression, is shown. Data are representative of independent biological experiments performed at least three times. **p < 0.01.

Src activity has been previously found to be involved in modulating cell signalling downstream of EGFR and participates in the regulation of cell proliferation.^42,43^ Moreover, EPS8 overexpression leads to enhanced cell proliferation and increased expression of multiple cell cycle regulators.^33^ Previous studies have documented constitutive phosphorylation of EPS8 in some cancer cell lines.^24^ Therefore, we determined the phosphorylation status of EPS8 in HN4 cells. As shown in Supplementary Fig. S1A, B, EPS8 is tyrosine phosphorylated in HN4 cells in the absence of growth factors. As EPS8 is a Src substrate,^30^ we sought to determine the impact of blocking Src phosphorylation of EPS8 on the expression of cell cycle mediators. Thus HN4 cells were first stably transfected with FFFF-EPS8 mutants, total cell lysates were prepared and the expression of FOXM1 and two of its targets, AURKA and AURKB, was determined by western blotting. As indicated in Fig. 1a–d, blocking EPS8 phosphorylation at all four sites (FFFF-EPS8 mutant) significantly reduced the expression of FOXM1, AURKA and AURKB compared with the non-transfected control, showing the importance of Src phosphorylation sites of EPS8 in controlling the expression of these cell cycle regulators. To determine whether blocking Src-mediated EPS8 phosphorylation is responsible for the reduced expression of cell cycle regulators, HN4 cells were treated with 400 nM of the Src inhibitor, dasatinib, for 24 h. Total cell lysates were prepared and the effect on cell cycle regulators was determined by western blotting. As shown in Fig. 1e–h, there was a significant reduction in the expression of FOXM1, AURKA and AURKB with dasatinib treatment relative to vehicle-treated controls. Furthermore, phosphorylation of EPS8 on tyrosine is reduced, but not completely abolished, by treatment with dasatinib (Supplementary Fig. S2A, B). In addition, the FFFF-EPS8 mutant shows substantially reduced tyrosine phosphorylation compared to the native protein even in the absence of dasatinib, based on the relative abundance of each in the cell (Supplementary Fig. S2C, D). Further reduction of tyrosine phosphorylation was observed in the FFFF-EPS8 protein upon dasatinib treatment and may indicate that additional tyrosines within EPS8 are dasatinib sensitive, either directly or indirectly (for example, Src may phosphorylate another EPS8 kinase). Alternatively, this may be related to effects of the drug on kinases other than Src, as it is known to have activity against Abl and Kit, although neither of these has, as yet, been reported to phosphorylate EPS8. Together, these observations suggest that blocking the four Src phosphorylation sites of EPS8 using either a pharmacological approach or gene mutation decreases the expression of downstream targets of EPS8 that regulate the cell cycle.

### Y602 in EPS8 is crucial for the expression of cell cycle regulators

**Fig. 2:**
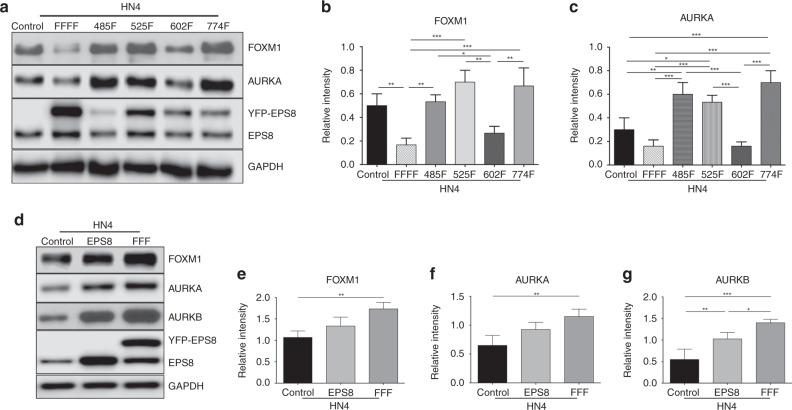
Contribution of individual Src phosphorylation sites in EPS8 on EPS8 downstream target expression. **a** HN4 cells stably transfected with EPS8 expression plasmids encoding FFFF, 485F, 525F, 602F and 774F mutants were used to prepare total cell lysates and then western blotted with the indicated antibodies. **b**, **c** Graphs indicate relative expression of FOXM1 and AURKA, in controls and mutant-expressing cells, normalised to GAPDH expression. Data shown are combined from three independent biological experiments. *p < 0.05, **p < 0.01, ***p < 0.001. **d** HN4 cells were stably transfected with an EPS8 expression plasmid or the FFF-EPS8 mutant. Total cell lysates were prepared, resolved by SDS-PAGE and western blotted with the indicated antibodies. **e**–**g** Graphs indicate relative expression of FOXM1, AURKA and AURKB, normalised to GAPDH expression. Data are representative of independent biological experiments performed on at least three separate occasions. *p < 0.05, **p < 0.01, ***p = 0.001.

To investigate the impact of Src-mediated phosphorylation of EPS8 at each single tyrosine residue (Y485, Y525, Y602 and Y774) on cell cycle regulation, HN4 cells were stably transfected with 485F-, 525F-, 602F- and 774F-EPS8 mutants, total cell lysates were prepared and the expression of FOXM1 and AURKA was determined by western blotting. As shown in Fig. 2a–c, cells expressing 602F-EPS8 showed a significant decrease in the expression of FOXM1 and AURKA compared to other single F-mutants, and at levels comparable with the FFFF-EPS8 mutant, suggesting that pY602 is a key regulatory phosphorylation within EPS8. To determine whether Y602 is indeed a crucial regulatory Src phosphorylation within EPS8, modulating cell cycle mediators, we generated stable transfectants of HN4 expressing FFF-EPS8 (blocking all Src phosphorylation sites except Y602). Total cell lysates were prepared and the expression of cell cycle mediators was determined by western blotting. As indicated in Fig. 2d–g, blocking EPS8 phosphorylation at Y485, Y525 and Y774 but not at Y602F (FFF-EPS8) significantly increased FOXM1, AURKA and AURKB expression compared with controls, similar to what was observed with overexpression of wild-type EPS8. The substantial reversion of EPS8 activity with the transition from FFFF-EPS8 to FFF-EPS8 mutant suggests that Y602 is a key phosphorylation site within EPS8 that regulates the expression of cell cycle mediators. Consistent with this, cells expressing the Y602F- or FFFF-EPS8 mutant showed a tendency to accumulate in G2/M (Supplementary Fig. S3).

### Blocking Src phosphorylation sites within EPS8 decreases cell proliferation

**Fig. 3:**
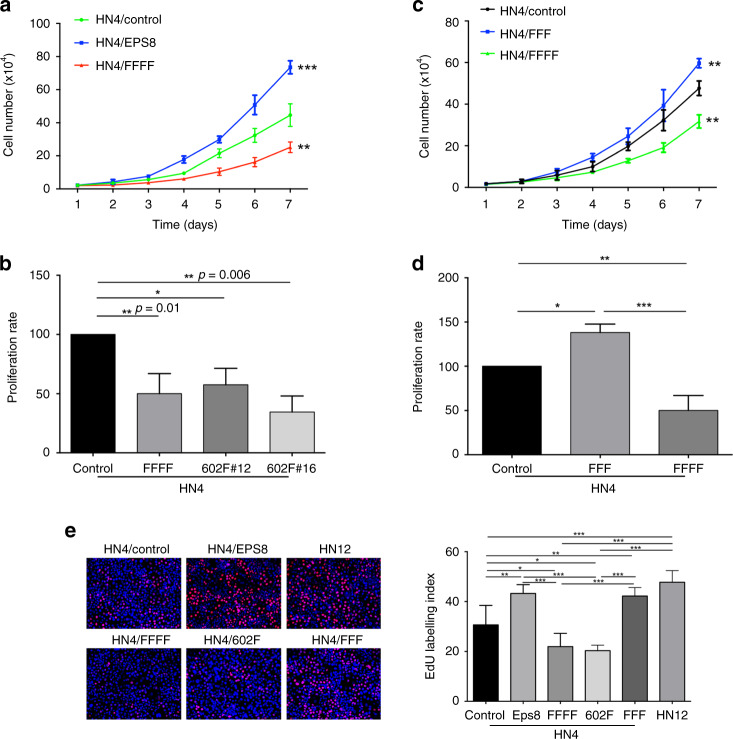
Blockade of Src phosphorylation sites in EPS8 alters cellular proliferation. **a** HN4 parental cells or overexpressing wild-type EPS8 or the FFFF-EPS8 mutant were plated in quadruplicate in 12-well culture plates at a density of 1 × 10^4^ cells per well, trypsinised and counted daily for 7 days, as indicated. Data shown are combined from independent biological experiments performed on three separate occasions. **p < 0.01, ***p < 0.001. **b** HN4 parental or HN4 cells expressing 602F-EPS8 (two different clones) or FFFF-EPS8 were plated in quadruplicate in multiwell plates. MTT reagent was added and solubilised after 1 and 7 days, and proliferation was calculated as described in “Methods”. Data shown are combined from experiments performed on three separate occasions. *p < 0.05. **c** HN4 parental cells or cells expressing FFF- and FFFF-EPS8 were plated in 12-well culture plates at a density of 1 × 10^4^ cells per well, then trypsinised and counted daily for 7 days, as indicated. Data shown are combined from experiments performed on three separate occasions. **p < 0.01. **d** HN4 parental, FFF- and FFFF-EPS8 expressing cells were plated in quadruplicate in multiwell plates, then MTT assays were performed after 1 and 7 days. Data shown are combined from three independent experiments. *p < 0.05, **p < 0.01, ***p < 0.001. **e** The indicated cells were seeded on coverslips, pulsed with EdU, stained, imaged at ×200 and counted as described in “Methods”. Data are representative of six microscope fields from triplicate coverslips and from two independent biological experiments. *p < 0.05, **p < 0.01, ***p < 0.001.

Previous studies have indicated that EPS8 overexpression leads to an increase in tumour cell proliferation.^32^ Therefore, we investigated whether Src-induced phosphorylation contributes to this function of EPS8. HN4 cells, with or without the expression of EPS8 mutants, were plated, and the cell numbers were determined by counting at 24-h intervals for 7 days. As shown in Fig. 3a, cells expressing the FFFF-EPS8 mutant showed a statistically significant decrease in proliferation compared to all controls, indicating that blocking the Src phosphorylation sites in EPS8 reduces cell proliferation. As a result of the data obtained above that showed blocking Y602 in EPS8 reduces the expression of cell cycle regulators, we sought to determine the impact of this mutation on tumour cell proliferation. Cells were plated and metabolically active cells were determined after 1 and 7 days by MTT assay. The data indicate that blocking Y602-EPS8 leads to a significant reduction in cell proliferation compared to the control, and the effect was comparable to that seen with the FFFF-EPS8 mutant (Fig. 3b), suggesting that phosphorylation of Y602 in EPS8 is crucial for proliferation of tumour cells in culture. To confirm whether blockade of Y602 is responsible for the reduction of tumour cell growth, we examined the differences in the effect of FFF- and FFFF-EPS8 mutants on tumour cell proliferation. HN4 cells expressing these mutants were plated, and the cell numbers were determined as previously over 7 days. As shown in Fig. 3c, cells expressing the FFF-EPS8 mutant showed enhanced proliferation compared to the control or cells expressing the FFFF-EPS8 mutant. Similar data were obtained by MTT assay (Fig. 3d). To complement these experiments, we also carried out EdU incorporation assays to determine the fraction of cells synthesising DNA. As shown in Fig. 3e, incorporation of EdU into cellular DNA was significantly reduced in cells expressing the Y602F- and FFFF-EPS8 mutants, whereas cells overexpressing the wild-type protein or the FFF-EPS8 mutant showed increased DNA synthesis. These data provide further evidence that phosphorylation of EPS8 at Y602 is crucial for regulating cell proliferation.

### Blocking Src phosphorylation sites of EPS8 decreases cell migration

**Fig. 4:**
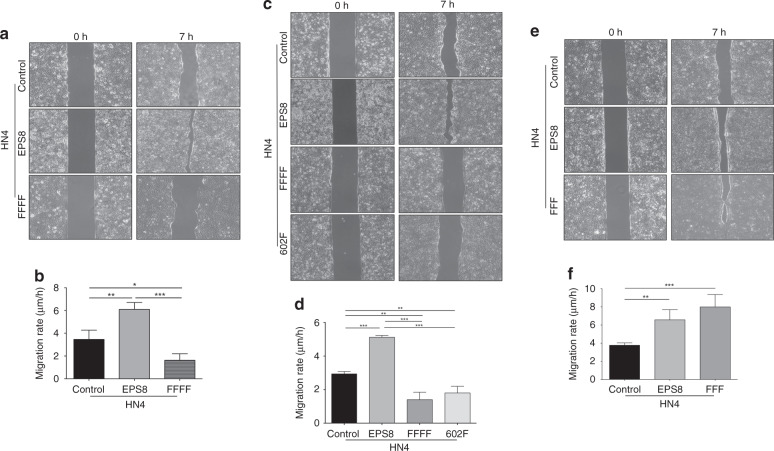
Impact of blocking Src phosphorylation sites of EPS8 on HN4 cell migration. **a** HN4 parental cells or those overexpressing wild-type EPS8 or FFFF-EPS8 were plated in culture inserts in triplicate and incubated overnight. Subsequently, the inserts were removed, and cells were allowed to migrate for 7 h. **b** Quantitation of cell migration. Data represent the combination from three independent experiments. *p < 0.01, **p < 0.001, ***p < 0.0001. **c** HN4 parental cells or cells overexpressing EPS8 or the FFFF- or 602F-EPS8 mutants were plated in triplicate in culture inserts and incubated overnight. Subsequently, the inserts were removed and cells were allowed to migrate for 7 h. **d** Quantitation of cell migration. Data represent the combination from three independent biological experiments. **p ≤ 0.001, ***p ≤ 0.0001. **e** HN4 parental cells or cells overexpressing EPS8 or the FFF-EPS8 mutant were plated in triplicate in culture inserts. Subsequently, the inserts were removed, and cells were allowed to migrate for 7 h. **f** Quantitation of cell migration. Data represent the combination from three independent biological experiments. **p = 0.01, ***p < 0.001.

Previous studies in our laboratory have shown that elevated expression of EPS8 contributes to increased migration of squamous carcinoma cells.^32^ Therefore, to test whether EPS8 phosphorylation has an impact on cell migration, we assayed the ability of cells expressing FFFF-EPS8 to migrate in a wound closure assay. As indicated in Fig. 4a, b, cells expressing the FFFF-EPS8 mutant showed a significant decrease in migration compared to controls. We extended our observations by testing whether expressing the 602F-EPS8 mutant was sufficient to alter the ability of HN4 cells to migrate. As shown in Fig. 4c, d, HN4–602F cells showed a significant decrease in migration compared to the controls, and this was comparable to that of cells expressing the FFFF-EPS8 mutant. These data suggest that blocking the four Src phosphorylation sites of EPS8 decreases cell motility and that, of these, pY602 is crucial for modulating cell migration. As the data described above (Fig. 3) showed a change in cell proliferation with the transition from FFFF- to FFF-EPS8 mutant, we extended our observations by testing whether HN4 cells show differential ability to migrate when expressing the FFF-EPS8 mutant. As shown in Fig. 4e, f, cells expressing the FFF-EPS8 mutant showed a significant increase in migration compared to controls. Taken together, the changes in tumour cell proliferation and migration with the transition from FFFF to FFF mutant suggest that Y602 is a crucial regulatory site within EPS8 that impacts the biological activity of HNSCC, consistent with effects on cell cycle regulators described above.

### Blocking Src phosphorylation sites in EPS8 reduces the expression of vimentin

**Fig. 5:**
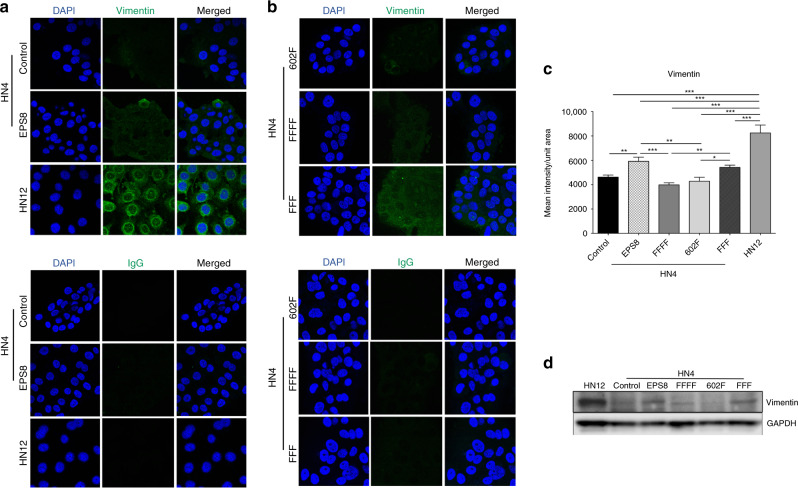
Src phosphorylation site mutants of EPS8 elicit changes in the vimentin expression. **a**, **b** HN4 parental cells or cells overexpressing wild-type EPS8, FFFF-, FFF- or 602F-EPS8 or HN12 cells as control were grown in triplicate on coverslips, fixed with cold methanol and immunostained with vimentin antibody or with mouse IgG as a negative control, followed by AlexaFluor488-conjugated secondary antibodies and counterstained with DAPI. Original magnification, ×400. **c** Quantitation of vimentin immunofluorescence. *p < 0.05, **p < 0.01, ***p < 0.0001. **d** Total cell lysates were prepared from the indicated cells and western blotted with vimentin and GAPDH antibodies, as indicated.

Oral squamous carcinoma cells expressing low levels of keratin, the major intermediate filament proteins in cells of epithelial origin, have a higher capacity to invade in vitro.^44^ Previously, we reported enhanced expression of vimentin in HN12 cells, derived from a synchronous lymph node metastasis in the patient from whom HN4 cells were derived,^45^ and that express relatively higher levels of EPS8.^46^ Furthermore, it has been reported that repression of EPS8 expression leads to reduced levels of vimentin in cervical cancer cells.^47^ Therefore, we sought to test whether mutation of the Src phosphorylation sites within EPS8 might play a role in regulating intermediate filament protein expression. We found no differences in keratin expression among EPS8 mutant-expressing HN4 cells (Supplementary Fig. S4). In contrast, ectopic expression of wild-type EPS8 or FFF-EPS8 produced a significant increase in vimentin expression in HN4 cells, as judged by both immunofluorescence and western blotting (Fig. 5a–d), but FFFF- and 602F-EPS8 did not. These data indicate a role for phosphorylation at Y602 in promoting a vimentin-positive phenotype of cancer cells and is consistent with the enhanced migration of these cells reported above.

### Impact of blocking Src phosphorylation sites of EPS8 on tumorigenesis

**Fig. 6:**
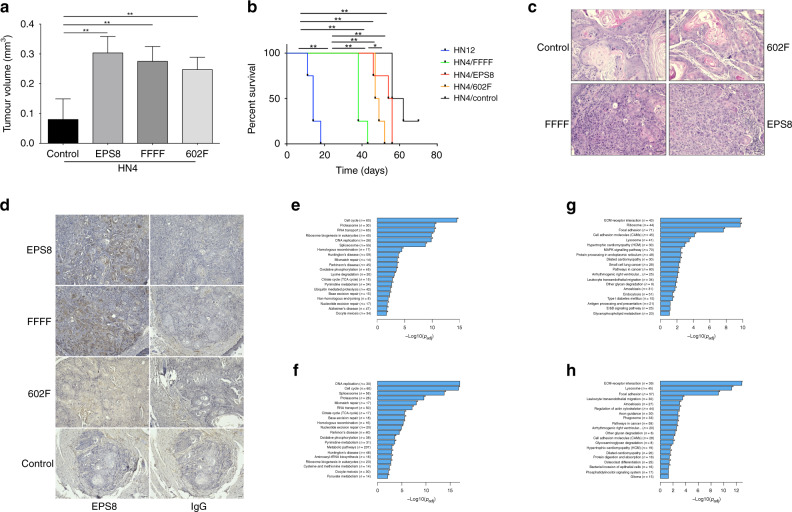
Src phosphorylation site mutants of EPS8 retain tumorigenic properties. **a** HN4 parental cells or cells overexpressing EPS8, FFFF- or 602F-EPS8 were trypsinised, counted and resuspended. In all, 1 × 10^5^ cells were injected orthotopically into the tongue or 5 × 10^4^ HN12 cells as control, and the mice monitored daily for 11 weeks, as described in “Methods”. Tumour volume differences in the tongue groups, **p < 0.01. **b** Kaplan–Meier overall survival curves of HN12 (blue line, *n* = 4), HN4/FFFF (green line, *n* = 4), HN4/EPS8 (red line, *n* = 4), HN4/602 F (orange line, *n* = 4), and HN4 parental (black line, *n* = 4). *p < 0.05, **p < 0.01. **c** Representative micrographs of haematoxylin and eosin-stained tongue tumour sections. Original magnification, ×200. Scale bar, 50 μm. **d** Tongue tumour sections were stained with EPS8 antibody or normal IgG as a control as described in “Methods”. Bar = 50 μm. **e**–**h** Total RNA was prepared from cell cultures and transcriptomic profiling was performed by RNA sequencing. **e** KEGG pathways showing significant downregulation in HN4/602F-EPS8 cells. **f** Pathways showing significant downregulation in dasatinib-treated HN4 cells. **g** Pathways showing significant upregulation in HN4/602F-EPS8 cells. **h** Pathways showing significant upregulation in dasatinib-treated HN4 cells.

Previously, our laboratory reported that overexpression of EPS8 correlated with an increasingly aggressive phenotype in vivo.^32^ Therefore, we determined whether blocking the Src phosphorylation sites within EPS8 would alter the tumorigenic properties of HN4 cells, which have inherently low tumorigenic potential. EPS8 mutant-expressing and control cells were injected into the tongues of athymic mice, and tumour formation was monitored. As indicated in Fig. 6a, cells overexpressing wild-type EPS8 formed significantly larger tumours than the control cells (around threefold). However, not only did FFFF-EPS8- and 602F-expressing cells form tumours of similar size to wild-type EPS8-expressing cells but also, strikingly, the latency of tumour formation and the survival time of the mice was significantly shorter for FFFF-EPS8-expressing cells compared to those overexpressing wild-type EPS8 (Fig. 6b), although not as abbreviated as with HN12 cells, which led to the death of all mice within the first 3 weeks and with half of the number of cells inoculated. In addition, tumours formed by cells expressing FFFF-EPS8 were less well differentiated compared to tumours formed by parental cells (Fig. 6c). Immunohistochemical analysis of EPS8 showed strong intratumoural expression in FFFF-EPS8 and HN4/EPS8 tumours, with much weaker expression in tumours generated from 602F-EPS8-expressing cells, and the expression was undetectable above background in the smaller tumours from the control cells (Fig. 6d). These data indicate that, although blocking the Src phosphorylation sites in EPS8 reduces proliferation and migration in cell culture, this does not appear to reduce, and actually enhances, tumorigenic potential in vivo.

### Blocking Src phosphorylation sites within EPS8 promotes signalling through alternative pro-oncogenic pathways

Given the somewhat unexpected result that cells expressing EPS8 phosphorylation site mutants were tumorigenic yet showed reduction in the in vitro parameters that we measured, we carried out whole-transcriptome sequencing studies to explore changes in gene expression occurring in these cells. Thus we compared gene expression in cells expressing 602F-EPS8, which showed reduced proliferation and migration in cell culture yet formed larger tumours than controls. As shown in Fig. 6e–h, Kyoto Encyclopedia of Genes and Genomes pathway analysis revealed differential enrichment for multiple functions upon comparing genes that were either downregulated or upregulated in cells expressing 602F-EPS8 compared to controls. Among the genes showing significant downregulation in 602F-EPS8-expressing cells were those in biological processes such as cell cycle, DNA replication and DNA repair, as well as metabolic processes involved in carbohydrate metabolism, including citric acid cycle and oxidative phosphorylation (Fig. 6e and Supplementary Table S1). Moreover, when we compared these results with those obtained following short-term (24 h) treatment of HN4 cells with dasatinib, there was considerable pathway overlap with 602F-EPS8-expressing cells (Fig. 6f and Supplementary Table S2). Conversely, transcripts showing upregulation in 602F-EPS8-expressing cells included those categorised into processes such as extracellular matrix (ECM)–receptor interaction, focal adhesion, cell adhesion molecules and mitogen-activated protein kinase (MAPK) signalling (Fig. 6g and Supplementary Table S3). Similar processes were enriched in dasatinib-treated cells (Fig. 6h and Supplementary Table S4). To begin to explore some of these changes, we determined ERK and AKT activation by western blotting. Supplementary Fig. S5 shows elevated AKT activity in 602F-EPS8-expressing cells compared to control, as judged by reactivity with pS^473^-AKT antibody, whereas both AKT and ERK activity were elevated in FFFF-EPS8-expressing cells. As PTEN is a negative regulator of PI3K/AKT signalling, we determined the levels of this phosphatase in cells expressing EPS8 mutants. As shown in Supplementary Fig. S6, expression remains relatively similar compared to levels in control cells, and thus reduction in PTEN is unlikely to explain the elevated AKT activity, with the caveat that PTEN function can be lost through mutation, although relatively rare in HNSCC.^48^ Taken together, the data suggest that, while blockade of EPS8 phosphorylation by Src may downregulate cell cycle-related and other genes important for proliferation in cell culture, there is concomitant upregulation of other genes, the products of which may serve to promote tumorigenesis in vivo.

## Discussion

EPS8 was first identified almost three decades ago,^20,49^ yet relatively little is known about how it is regulated to mediate its downstream signalling functions, although it has previously been reported to be constitutively tyrosine phosphorylated in some cancer cells,^23^ including those transformed by v-src.^38^ In this study, therefore, we investigated the impact of blocking four tyrosine residues within EPS8, previously reported as targets for Src activity,^30^ on the expression of downstream mediators of the cell cycle and associated biological properties. For these studies, we used HN4 squamous carcinoma cells, derived from a primary tongue tumour and that we have previously characterised as expressing similar levels of EPS8 to normal keratinocytes. HN4 cells are poorly tumorigenic and show substantially reduced migratory properties compared to HN12 cells isolated from a synchronous lymph node metastasis in the same patient, express threefold higher levels of EPS8 and are highly invasive and tumorigenic.^32,39,46^ We found that, whereas mutating all four sites together to phenylalanine led to reduced expression of FOXM1 and its target genes AURKA and AURKB, a similar result was obtained when only Y602 was mutated but not with any of the other four single substitutions at residues 485, 525 or 774. Moreover, cells expressing the triple (485/525/774) phenylalanine-substituted EPS8 actually showed elevated expression of these downstream mediators, similar to that seen in cells overexpressing the unmodified protein. These results indicate that phosphorylation at Y602 is likely crucial for the ability of EPS8 to regulate FOXM1 expression. Consistent with our previous studies that identified FOXM1 as a key mediator of cell proliferation and migration downstream of EPS8,^33^ we found that cells expressing 602F-EPS8 (and also the quadra-substituted mutant) grew significantly more slowly and failed to migrate as fast as the controls, whereas the triple-substituted mutant showed the opposite properties. Thus phosphorylation at Y602 of EPS8, likely by Src, should act to promote cell cycle progression as well as a migratory phenotype.

The critical regulatory impact of phosphorylation of EPS8 at Y602 seems clear, based on our current data, yet the mechanism through which this effect is realised is currently unknown. EPS8 is known to bind multiple protein partners within the cell, principally through interactions between proline-rich regions and SH3 domains, as EPS8 contains both of these. One possibility to explain the crucial nature of pY602 is the location of the target site, as it is situated between the SH3 domain (residues 531–590) and the main proline-rich region (residues 617–646). Thus phosphorylation at Y602 may serve to induce a conformational change in EPS8 and/or modify the ability of EPS8 to bind to other proteins either through its SH3 domain or its proline-rich region. Using the phenylalanine-substituted mutants described above, we are actively investigating this possibility.

The acquisition of partial mesenchymal characteristics has been associated with more aggressive behaviour of squamous carcinoma cells,^45,50–52^ such as enhanced motility and tumorigenic potential. In previous studies, in which we reduced the expression of EPS8 by RNA interference in vimentin-positive SCC cells, we found not only a reduction in motility and tumorigenicity but also the acquisition of a more differentiated phenotype in the small tumours that were formed.^32^ When we investigated the impact of modifying the Src phosphorylation sites within EPS8 in the present study, we found that vimentin expression was increased in cells overexpressing unmodified EPS8 or the triple phenylalanine-substituted version, but not 602F-EPS8 or the quadra-substituted protein. This is consistent with Src-dependent phosphorylation of EPS8 playing at least a partial regulatory role in the process of epithelial-to-mesenchymal transition. Several reports exist of a relationship between Src and EMT in cancer.^53^ In colon cancer cells, Src disrupts the localisation of E-cadherin and the expression of vimentin, and this is dependent upon integrin signalling and the ability of Src to phosphorylate focal adhesion kinase,^54^ with accumulation of phosphomyosin at the cell periphery.^55^ Elevated vimentin expression related to increased EPS8 has been reported previously in cervical cancer cells.^47^ The results of our current study provide evidence that not only is EPS8 overexpression related to upregulation of vimentin but also its phosphorylation by Src at Y602 is required, thus providing another link between Src activity and the promotion of EMT in carcinoma cells. It is, however, unlikely that EPS8 functions as the sole switch to trigger EMT. Although EPS8 overexpression or modification may lead to changes in the expression of vimentin and enhance the migratory phenotype, as indicated in this study and previously by others, acquisition of full mesenchymal characteristics will, undoubtedly, require additional signalling events.

Although we had previously found HN4/EPS8 cells to be tumorigenic in orthotopic assays,^32^ the finding in this study that cells expressing either 602F-EPS8 or the quadra-substituted EPS8 were highly tumorigenic, compared to controls, was surprising to us, given the reduced proliferation and motility that we observed in cell culture. Although the reasons for this remain to be established experimentally, some insight may be gleaned from the transcriptomic studies that we carried out. As might be predicted from the behaviour of these cells in culture, cells expressing 602F-EPS8 showed significant downregulation of a cohort of genes whose products are required for cell cycle progression and DNA replication. However, there was significant upregulation of genes related to multiple processes that might drive tumorigenesis in vivo, such as those involved in ECM–receptor interaction, focal adhesion and cell adhesion molecules, as well as MAPK signalling. In addition, in cells expressing 602F-EPS8, mediators of the tricarboxylic acid cycle and oxidative phosphorylation were diminished, which may indicate a shift to aerobic glycolysis. Comparing the biological processes that were significantly enriched in 602F-EPS8-expressing cells with those in dasatinib-treated cells compared to control cells, there were considerable similarities in the downregulated processes, including cell cycle, replication and DNA repair, as well as in the processes that were upregulated under these conditions such as ECM–receptor interaction, focal adhesion and others. These observations enhance confidence that the results obtained with 602F-EPS8 are representative of conditions where EPS8 is not phosphorylated by Src.

Although inhibiting Src has been effective in some preclinical studies, the efficacy of dasatinib in clinical trials thus far has been disappointing. Patients with RM HNSCC treated with dasatinib showed no objective response, in spite of its ability to block Src kinase activity.^56^ Moreover, in a randomised trial of patients with operable HNSCC, dasatinib showed neither efficacy as a single agent nor any additive effect when combined with a tyrosine kinase inhibitor.^57^ Based on the data in the current study, it may be that tumour cells can switch to activate alternative pathways upon treatment with dasatinib and that it may be related, at least in part, to the action of Src on EPS8. Thus altered signalling such as this may afford additional opportunities for more efficacious targeted therapeutic intervention.

In summary, we have demonstrated that modification of EPS8 at Src target sites plays a key role in downstream signalling and tumour biology. In particular, a key finding of this study is that Y602 is a crucial regulatory Src phosphorylation site within EPS8 that impacts its biochemical and biological functions in HNSCC. Our data suggest that blockade of EPS8 phosphorylation by Src may trigger alternative signal transduction pathways, enabling cells to survive and grow, underpinning resistance to dasatinib.

## Supplementary information

Supplementary Information

## Data Availability

All data generated or analysed during this study are included in this published article [and its supplementary information files].

## References

[1] Rothenberg SM, Ellisen LW (2012). The molecular pathogenesis of head and neck squamous cell carcinoma. J. Clin. Invest..

[2] Parkin DM, Bray F, Ferlay J, Pisani P (2005). Global cancer statistics, 2002. CA Cancer J. Clin..

[3] Sacco AG, Cohen EE (2015). Current treatment options for recurrent or metastatic head and neck squamous cell carcinoma. J. Clin. Oncol..

[4] Price KA, Cohen EE (2012). Current treatment options for metastatic head and neck cancer. Curr. Treat. Options Oncol..

[5] Shahoumi LA, Yeudall WA (2019). Targeted therapies for non-HPV-related head and neck cancer: challenges and opportunities in the context of predictive, preventive, and personalized medicine. EPMA J..

[6] Bobdey S, Sathwara J, Jain A, Saoba S, Balasubramaniam G (2018). Squamous cell carcinoma of buccal mucosa: an analysis of prognostic factors. South Asian J. Cancer.

[7] Loeffler-Ragg J, Schwentner I, Sprinzl GM, Zwierzina H (2008). EGFR inhibition as a therapy for head and neck squamous cell carcinoma. Expert Opin. Investig. Drugs.

[8] Horn D, Hess J, Freier K, Hoffmann J, Freudlsperger C (2015). Targeting EGFR-PI3K-AKT-mTOR signaling enhances radiosensitivity in head and neck squamous cell carcinoma. Expert Opin. Ther. Targets.

[9] Braunholz D, Saki M, Niehr F, Ozturk M, Borras Puertolas B, Konschak R (2016). Spheroid culture of head and neck cancer cells reveals an important role of EGFR signalling in anchorage independent survival. PLoS ONE.

[10] Ang KK, Berkey BA, Tu X, Zhang HZ, Katz R, Hammond EH (2002). Impact of epidermal growth factor receptor expression on survival and pattern of relapse in patients with advanced head and neck carcinoma. Cancer Res..

[11] Herbst RS, Langer CJ (2002). Epidermal growth factor receptors as a target for cancer treatment: the emerging role of IMC-C225 in the treatment of lung and head and neck cancers. Semin. Oncol..

[12] Chung CH, Ely K, McGavran L, Varella-Garcia M, Parker J, Parker N (2006). Increased epidermal growth factor receptor gene copy number is associated with poor prognosis in head and neck squamous cell carcinomas. J. Clin. Oncol..

[13] Hynes NE, Lane HA (2005). ERBB receptors and cancer: the complexity of targeted inhibitors. Nat. Rev. Cancer.

[14] Testa JR, Bellacosa A (2001). AKT plays a central role in tumorigenesis. Proc. Natl Acad. Sci. USA.

[15] Vivanco I, Sawyers CL (2002). The phosphatidylinositol 3-Kinase AKT pathway in human cancer. Nat. Rev. Cancer.

[16] Lewis TS, Shapiro PS, Ahn NG (1998). Signal transduction through MAP kinase cascades. Adv. Cancer Res..

[17] Dowlati A, Nethery D, Kern JA (2004). Combined inhibition of epidermal growth factor receptor and JAK/STAT pathways results in greater growth inhibition in vitro than single agent therapy. Mol. Cancer Ther..

[18] Xie Z, Chen Y, Liao EY, Jiang Y, Liu FY, Pennypacker SD (2010). Phospholipase C-gamma1 is required for the epidermal growth factor receptor-induced squamous cell carcinoma cell mitogenesis. Biochem. Biophys. Res. Commun..

[19] Castagnino P, Biesova Z, Wong WT, Fazioli F, Gill GN, Di Fiore PP (1995). Direct binding of eps8 to the juxtamembrane domain of EGFR is phosphotyrosine- and SH2-independent. Oncogene.

[20] Fazioli F, Minichiello L, Matoska V, Castagnino P, Miki T, Wong WT (1993). Eps8, a substrate for the epidermal growth factor receptor kinase, enhances EGF-dependent mitogenic signals. EMBO J..

[21] Kishan KV, Scita G, Wong WT, Di Fiore PP, Newcomer ME (1997). The SH3 domain of Eps8 exists as a novel intertwined dimer. Nat. Struct. Biol..

[22] Tocchetti A, Confalonieri S, Scita G, Di Fiore PP, Betsholtz C (2003). In silico analysis of the EPS8 gene family: genomic organization, expression profile, and protein structure. Genomics.

[23] Matoskova B, Wong WT, Nomura N, Robbins KC, Di Fiore PP (1996). RN-tre specifically binds to the SH3 domain of eps8 with high affinity and confers growth advantage to NIH3T3 upon carboxy-terminal truncation. Oncogene.

[24] Matoskova B, Wong WT, Salcini AE, Pelicci PG, Di Fiore PP (1995). Constitutive phosphorylation of eps8 in tumor cell lines: relevance to malignant transformation. Mol. Cell. Biol..

[25] Karlsson T, Songyang Z, Landgren E, Lavergne C, Di Fiore PP, Anafi M (1995). Molecular interactions of the Src homology 2 domain protein Shb with phosphotyrosine residues, tyrosine kinase receptors and Src homology 3 domain proteins. Oncogene.

[26] Biesova Z, Piccoli C, Wong WT (1997). Isolation and characterization of e3B1, an Eps8 binding protein that regulates cell growth. Oncogene.

[27] Lanzetti L, Rybin V, Malabarba MG, Christoforidis S, Scita G, Zerial M (2000). The Eps8 protein coordinates EGF receptor signalling through Rac and trafficking through Rab5. Nature.

[28] Disanza A, Carlier MF, Stradal TE, Didry D, Frittoli E, Confalonieri S (2004). Eps8 controls actin-based motility by capping the barbed ends of actin filaments. Nat. Cell Biol..

[29] Scita G, Nordstrom J, Carbone R, Tenca P, Giardina G, Gutkind S (1999). EPS8 and E3B1 transduce signals from Ras to Rac. Nature.

[30] Cunningham DL, Creese AJ, Auciello G, Sweet SM, Tatar T, Rappoport JZ (2013). Novel binding partners and differentially regulated phosphorylation sites clarify Eps8 as a multi-functional adaptor. PLoS ONE.

[31] Maa MC, Lai JR, Lin RW, Leu TH (1999). Enhancement of tyrosyl phosphorylation and protein expression of eps8 by v-Src. Biochim. Biophys. Acta.

[32] Wang H, Patel V, Miyazaki H, Gutkind JS, Yeudall WA (2009). Role for EPS8 in squamous carcinogenesis. Carcinogenesis.

[33] Wang H, Teh MT, Ji Y, Patel V, Firouzabadian S, Patel AA (2010). EPS8 upregulates FOXM1 expression, enhancing cell growth and motility. Carcinogenesis.

[34] Frame MC (2004). Newest findings on the oldest oncogene; how activated src does it. J. Cell Sci..

[35] Summy JM, Gallick GE (2003). Src family kinases in tumor progression and metastasis. Cancer Metastasis Rev..

[36] Zhang J, Wang S, Jiang B, Huang L, Ji Z, Li X (2017). c-Src phosphorylation and activation of hexokinase promotes tumorigenesis and metastasis. Nat. Commun..

[37] Dai H, Lv YF, Yan GN, Meng G, Zhang X, Guo QN (2016). RanBP9/TSSC3 complex cooperates to suppress anoikis resistance and metastasis via inhibiting Src-mediated Akt signaling in osteosarcoma. Cell Death Dis..

[38] Leu TH, Yeh HH, Huang CC, Chuang YC, Su SL, Maa MC (2004). Participation of p97Eps8 in Src-mediated transformation. J. Biol. Chem..

[39] Yeudall WA, Miyazaki H, Ensley JF, Cardinali M, Gutkind JS, Patel V (2005). Uncoupling of epidermal growth factor-dependent proliferation and invasion in a model of squamous carcinoma progression. Oral Oncol..

[40] Jakus J, Yeudall WA (1996). Growth inhibitory concentrations of EGF induce p21 (WAF1/Cip1) and alter cell cycle control in squamous carcinoma cells. Oncogene.

[41] Patel V, Ensley JF, Gutkind JS, Yeudall WA (2000). Induction of apoptosis in head-and-neck squamous carcinoma cells by gamma-irradiation and bleomycin is p53-independent. Int. J. Cancer.

[42] Xi S, Zhang Q, Dyer KF, Lerner EC, Smithgall TE, Gooding WE (2003). Src kinases mediate STAT growth pathways in squamous cell carcinoma of the head and neck. J. Biol. Chem..

[43] Riley D, Carragher NO, Frame MC, Wyke JA (2001). The mechanism of cell cycle regulation by v-Src. Oncogene.

[44] Crowe DL, Milo GE, Shuler CF (1999). Keratin 19 downregulation by oral squamous cell carcinoma lines increases invasive potential. J. Dent. Res..

[45] Paccione RJ, Miyazaki H, Patel V, Waseem A, Gutkind JS, Zehner ZE (2008). Keratin down-regulation in vimentin-positive cancer cells is reversible by vimentin RNA interference, which inhibits growth and motility. Mol. Cancer Ther..

[46] Miyazaki H, Patel V, Wang H, Ensley JF, Gutkind JS, Yeudall WA (2006). Growth factor-sensitive molecular targets identified in primary and metastatic head and neck squamous cell carcinoma using microarray analysis. Oral Oncol..

[47] Li Q, Bao W, Fan Q, Shi WJ, Li ZN, Xu Y (2016). Epidermal growth factor receptor kinase substrate 8 promotes the metastasis of cervical cancer via the epithelial-mesenchymal transition. Mol. Med. Rep..

[48] Stransky N, Egloff AM, Tward AD, Kostic AD, Cibulskis K, Sivachenko A (2011). The mutational landscape of head and neck squamous cell carcinoma. Science.

[49] Fazioli F, Bottaro DP, Minichiello L, Auricchio A, Wong WT, Segatto O (1992). Identification and biochemical characterization of novel putative substrates for the epidermal growth factor receptor kinase. J. Biol. Chem..

[50] Han G, Lu SL, Li AG, He W, Corless CL, Kulesz-Martin M (2005). Distinct mechanisms of TGF-beta1-mediated epithelial-to-mesenchymal transition and metastasis during skin carcinogenesis. J. Clin. Invest..

[51] Kupferman ME, Jiffar T, El-Naggar A, Yilmaz T, Zhou G, Xie T (2010). TrkB induces EMT and has a key role in invasion of head and neck squamous cell carcinoma. Oncogene.

[52] Mandal M, Myers JN, Lippman SM, Johnson FM, Williams MD, Rayala S (2008). Epithelial to mesenchymal transition in head and neck squamous carcinoma: association of Src activation with E-cadherin down-regulation, vimentin expression, and aggressive tumor features. Cancer.

[53] Avizienyte E, Brunton VG, Fincham VJ, Frame MC (2005). The SRC-induced mesenchymal state in late-stage colon cancer cells. Cells Tiss. Org..

[54] Avizienyte E, Wyke AW, Jones RJ, McLean GW, Westhoff MA, Brunton VG (2002). Src-induced de-regulation of E-cadherin in colon cancer cells requires integrin signalling. Nat. Cell Biol..

[55] Avizienyte E, Fincham VJ, Brunton VG, Frame MC (2004). Src SH3/2 domain-mediated peripheral accumulation of Src and phospho-myosin is linked to deregulation of E-cadherin and the epithelial-mesenchymal transition. Mol. Biol. Cell..

[56] Brooks HD, Glisson BS, Bekele BN, Johnson FM, Ginsberg LE, El-Naggar A (2011). Phase 2 study of dasatinib in the treatment of head and neck squamous cell carcinoma. Cancer.

[57] Bauman JE, Duvvuri U, Gooding WE, Rath TJ, Gross ND, Song J (2017). Randomized, placebo-controlled window trial of EGFR, Src, or combined blockade in head and neck cancer. JCI Insight.

