# Supplementary material for: EPS8 phosphorylation by Src modulates its oncogenic functions
Source: Br J Cancer. 2020 Jul 9;123(7):1078–88. doi: 10.1038/s41416-020-0976-6 (PMC7525440; doi:10.1038/s41416-020-0976-6)
Supplement: Supplementary file 1 — Supplementary Information [file 41416_2020_976_MOESM1_ESM.pdf]

Fig. S1.

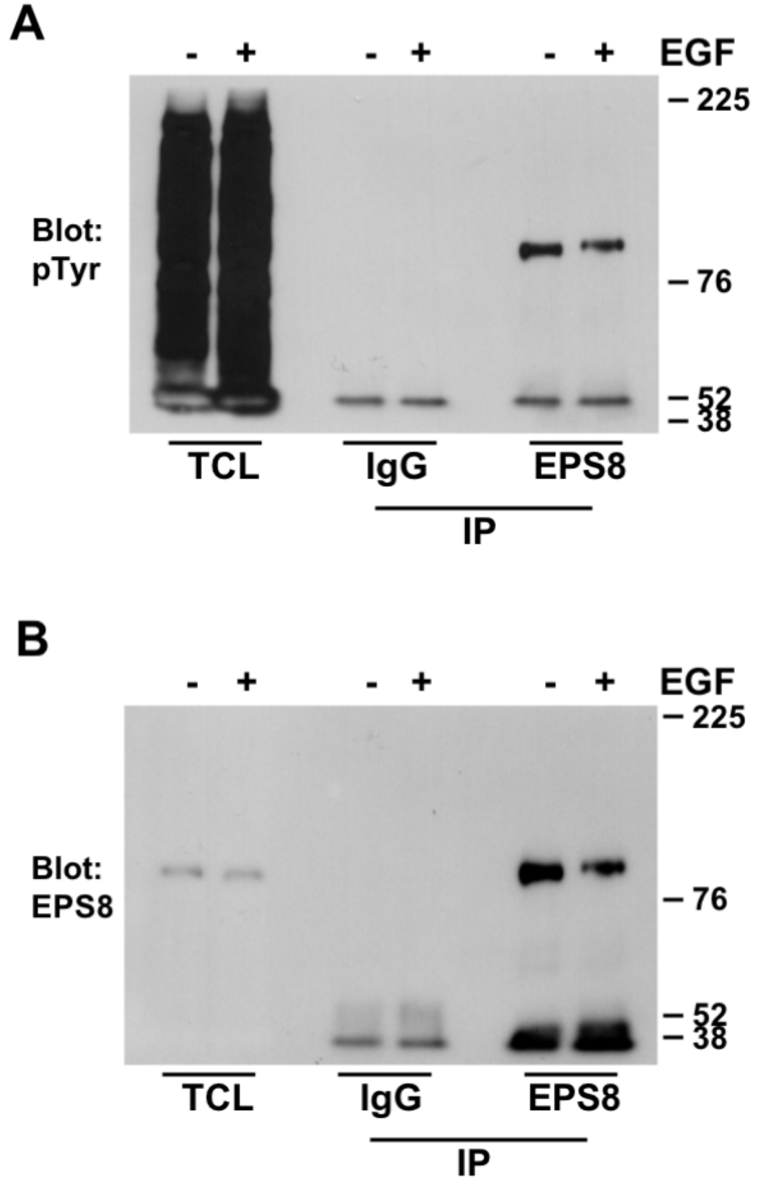
A
- + - + - + EGF
225
Blot:
pTyr
76
52
38
TCL
IgG
EPS8
IP
B
- + - + - + EGF
225
Blot:
EPS8
76
52
38
TCL
IgG
EPS8
IP

Fig. S2.

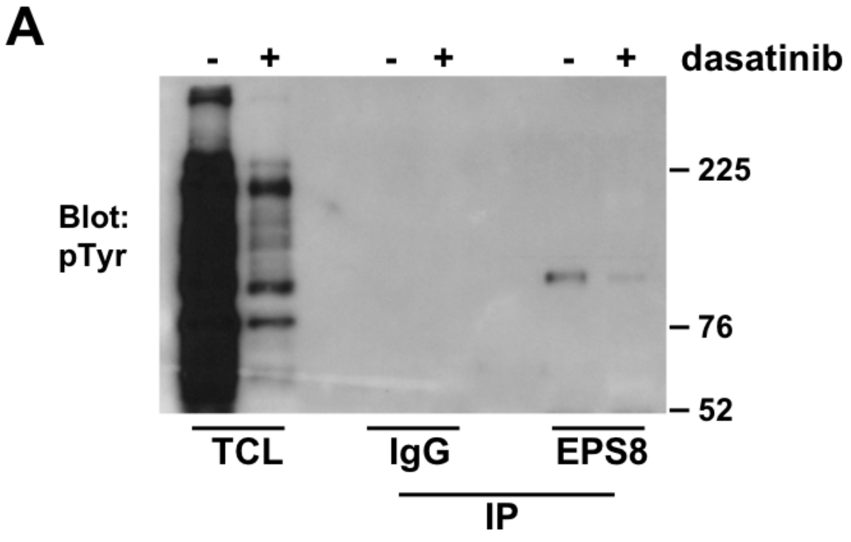

A
- + - + - + dasatinib
Blot:
pTyr
225
76
52
TCL
IgG
EPS8
IP

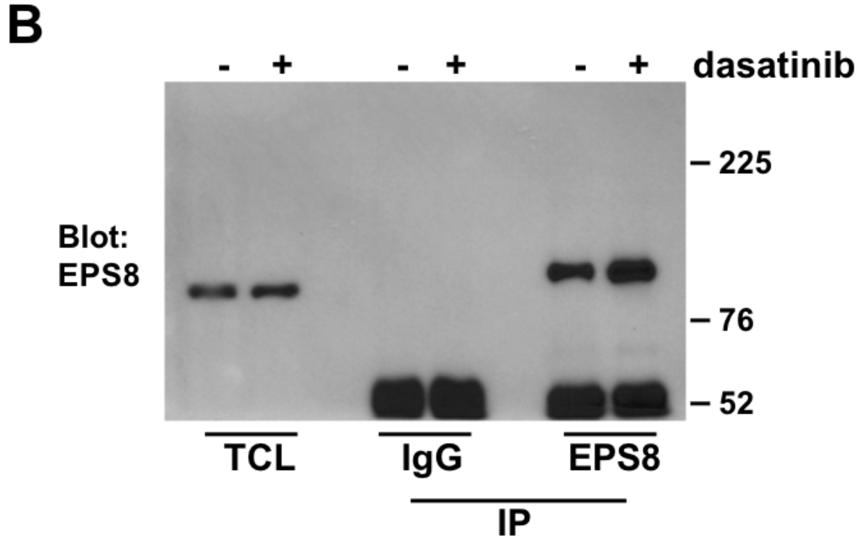

B
- + - + - + dasatinib
Blot:
EPS8
225
76
52
TCL
IgG
EPS8
IP

Fig. S2.

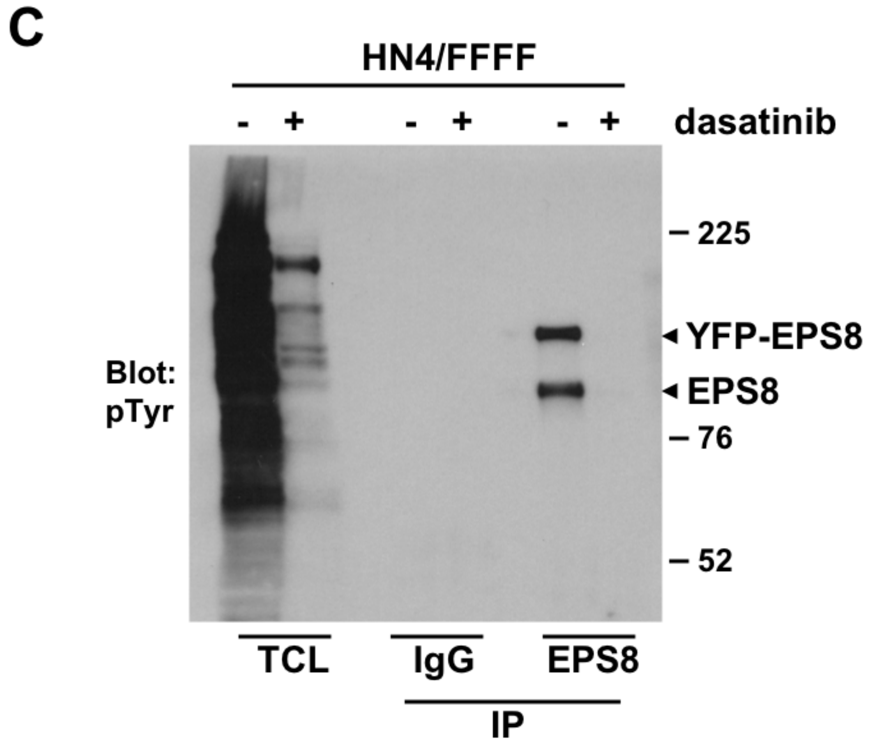
C
HN4/FFFF
- + - + - + dasatinib
225
YFP-EPS8
EPS8
76
52
Blot: pTyr
TCL
IgG
EPS8
IP

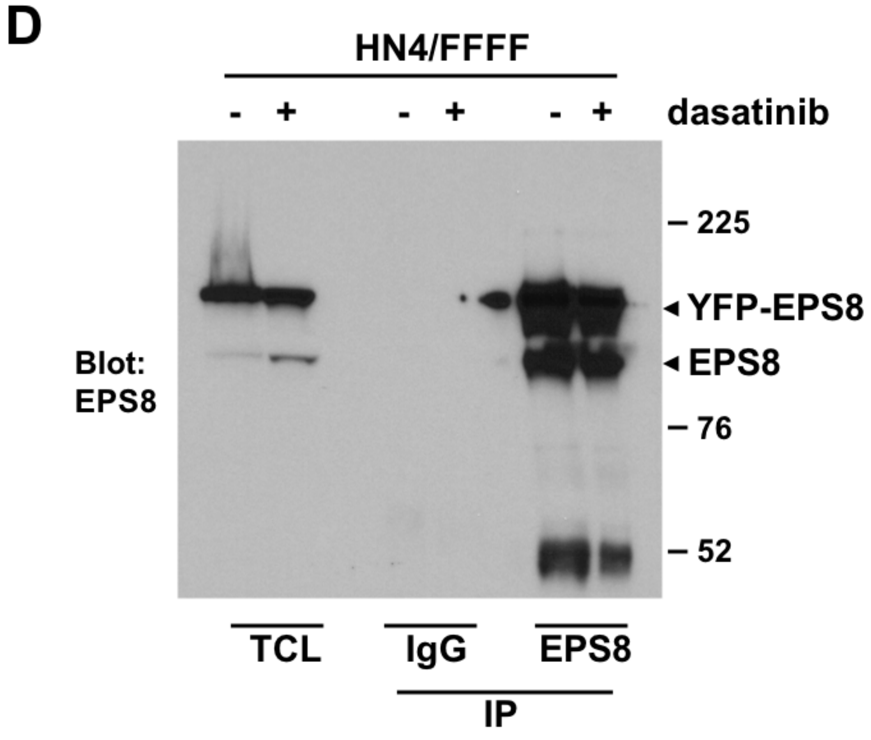
D
HN4/FFFF
- + - + - + dasatinib
225
YFP-EPS8
EPS8
76
52
Blot: EPS8
TCL
IgG
EPS8
IP

Fig. S3

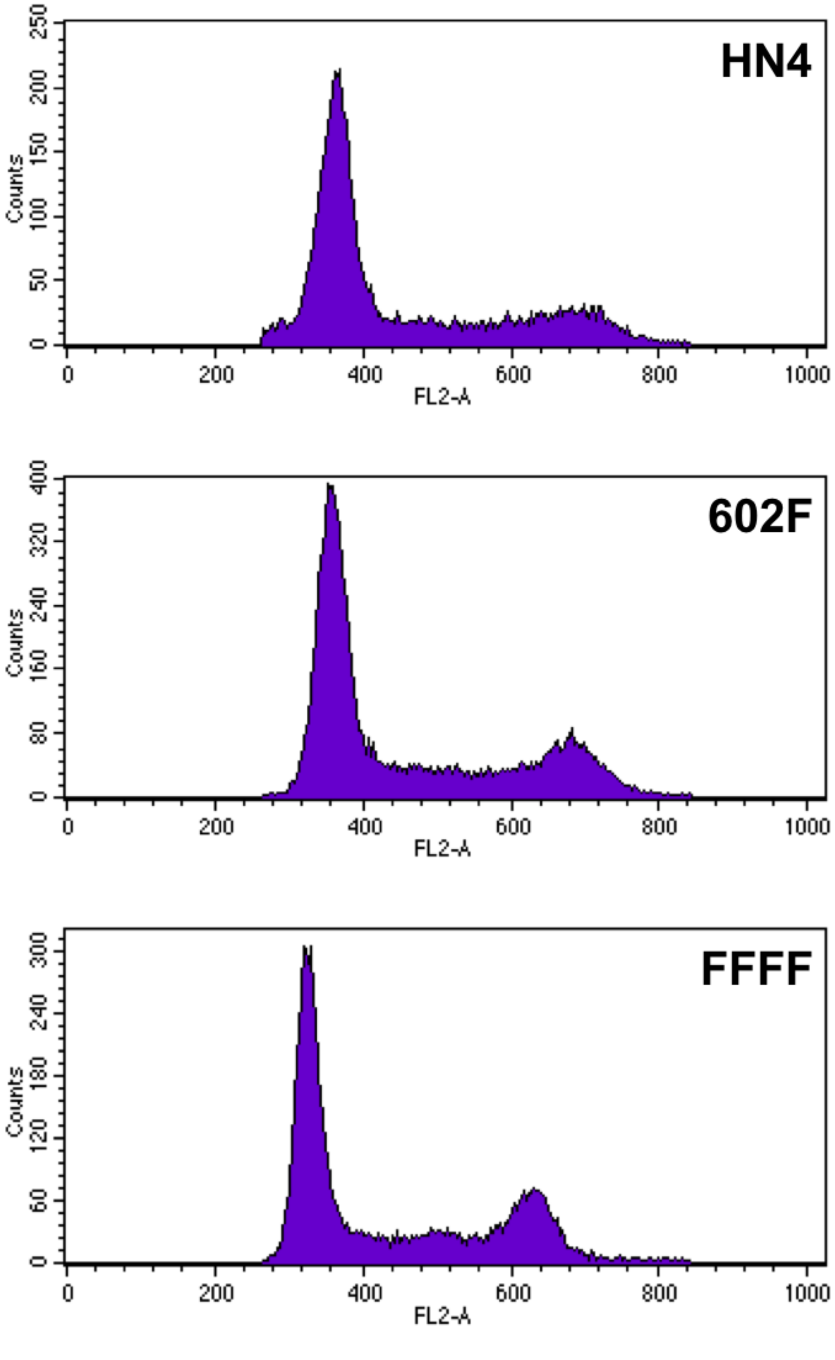

Cell Line	G1 (%)	S (%)	G2/M (%)
HN4	63.7	16.2	15.6
602F	61.3	17.4	18.4
FFFF	58.2	14.7	23

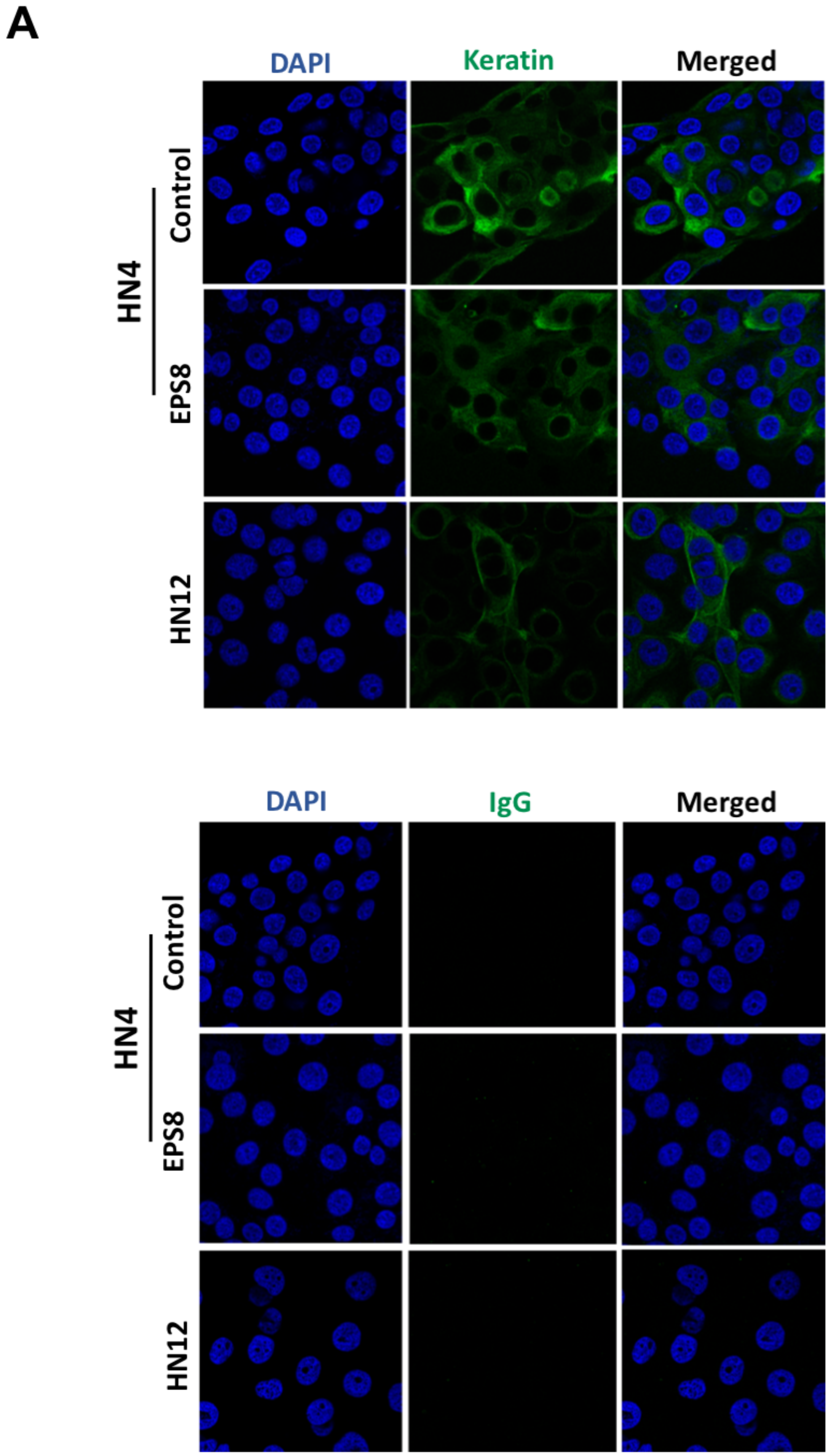
A
DAPI
Keratin
Merged
HN4
Control
EPS8
HN12
DAPI
IgG
Merged
HN4
Control
EPS8
HN12

Fig. S4.

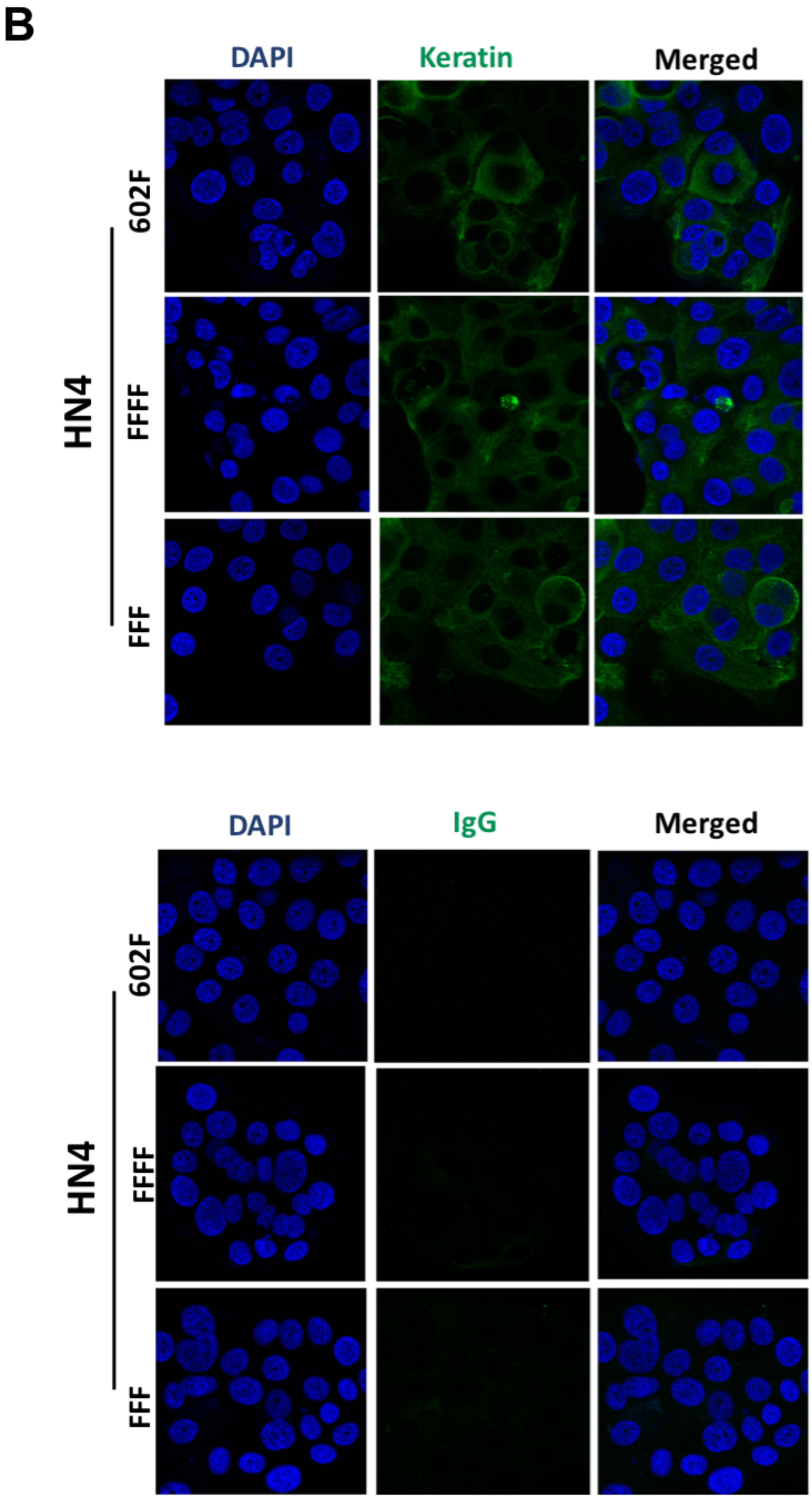
B
DAPI
Keratin
Merged
HN4
602F
FFFF
FFF
DAPI
IgG
Merged
HN4
602F
FFFF
FFF

Fig. S4.

C

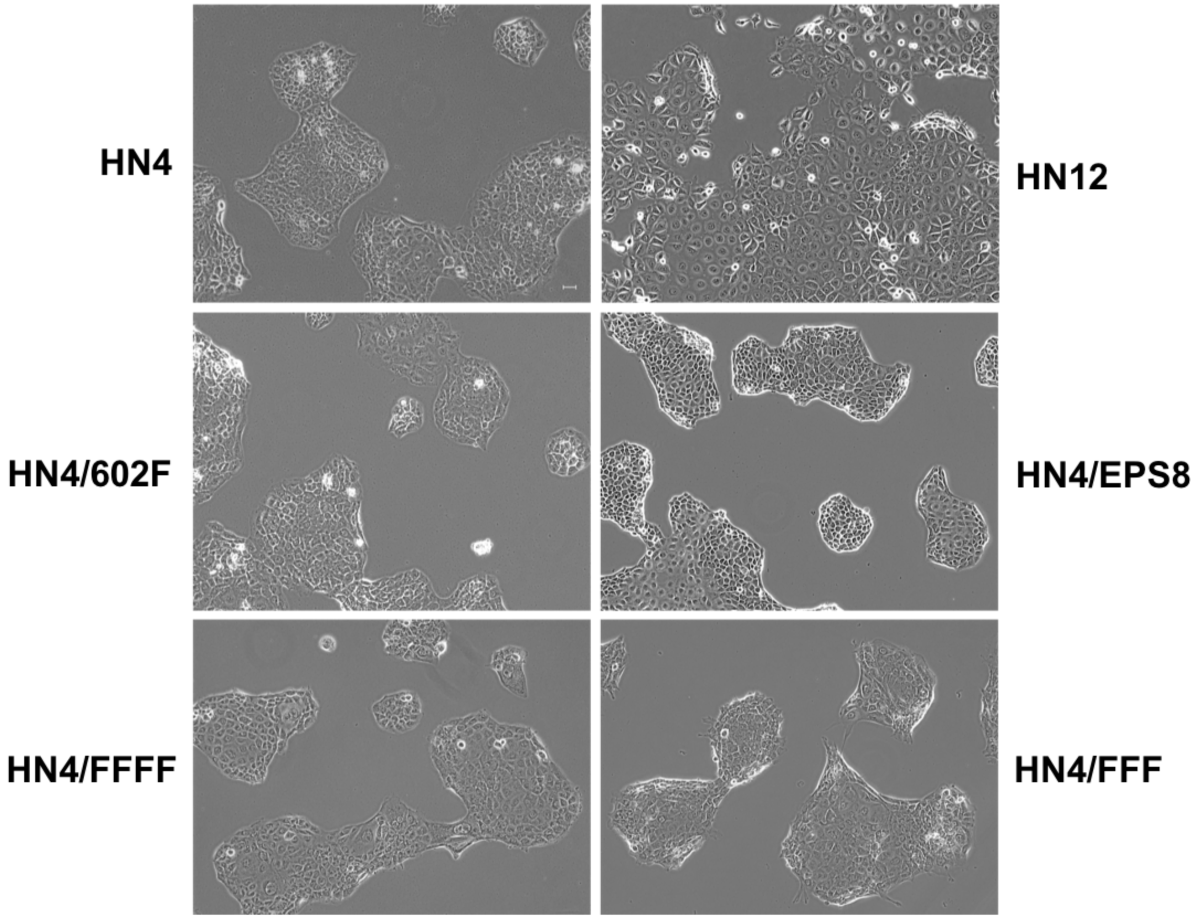

Fig. S4.

Fig. S5

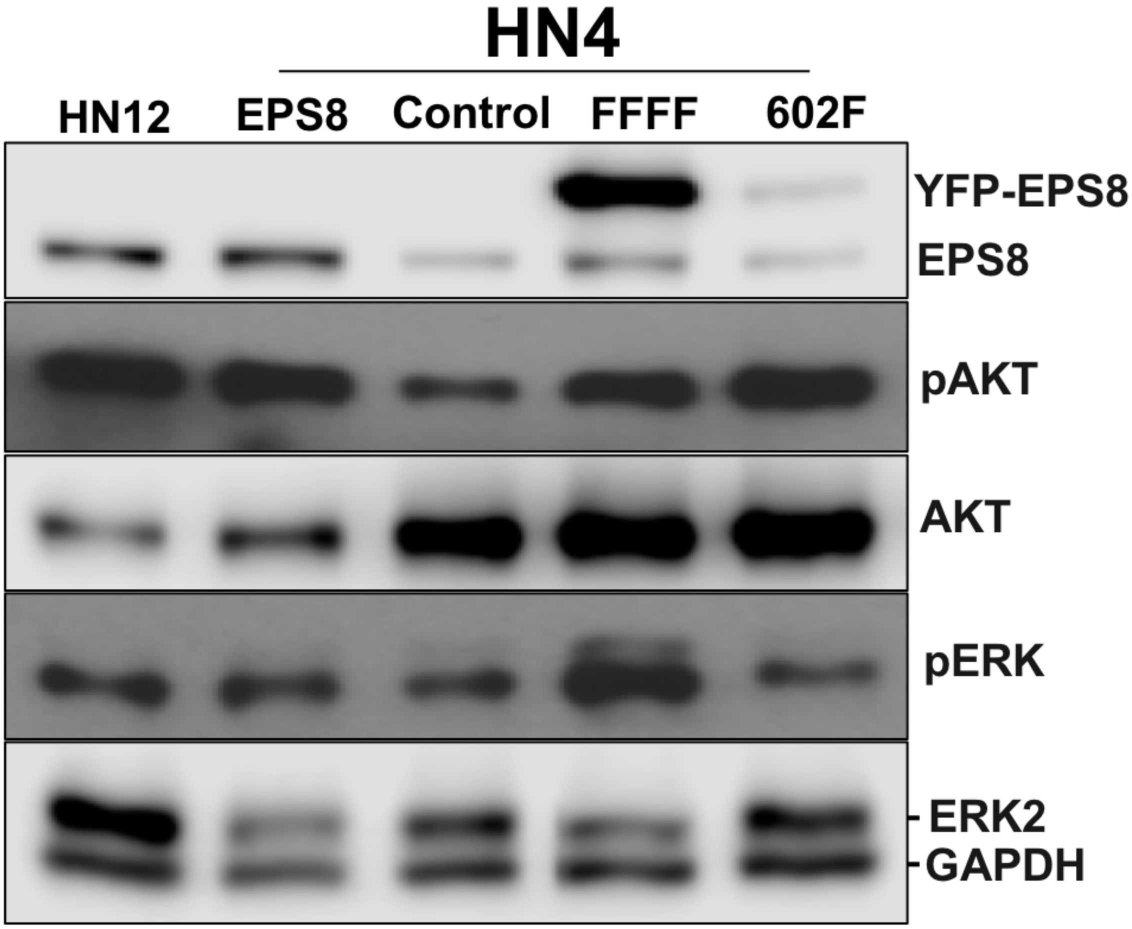
HN4
HN12
EPS8
Control
FFFF
602F
YFP-EPS8
EPS8
pAKT
AKT
pERK
ERK2
GAPDH

Fig. S6.

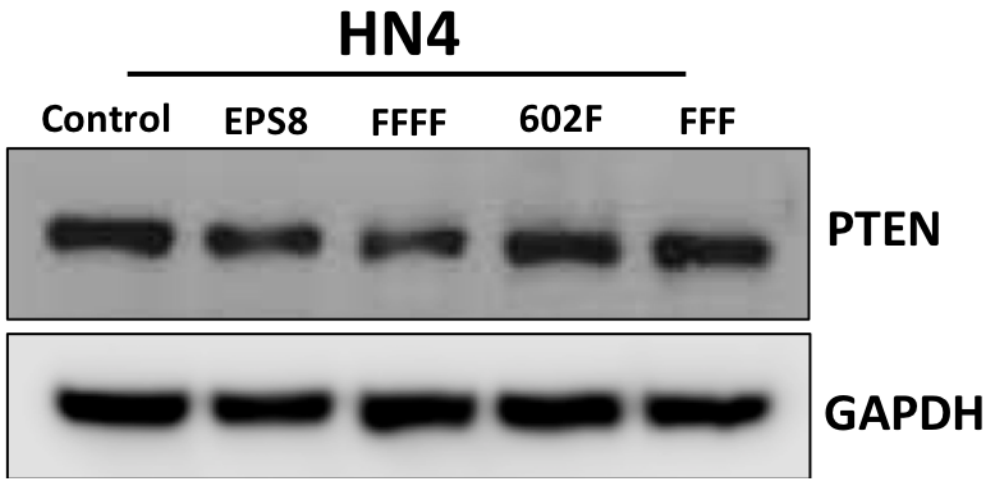

Legends to Supplementary Figures:

Figure S1: EPS8 is constitutively tyrosine-phosphorylated in HN4 cells. HN4 cells were deprived of serum for 16 h, and then lysed with or without addition of 10 ng/ml EGF for 10 min. EPS8 was immunoprecipitated as described in Methods and proteins resolved by SDS-PAGE and western blotted with antibodies that recognize phosphotyrosine (A). Antibody was then removed and the blot incubated with EPS8 antibody (B). TCL, 50 µg total cell lysate (5% input).

Figure S2: Dasatinib reduces tyrosine phosphorylation of EPS8. A. & B. HN4 cells were treated with 400 nM dasatinib, or an equivalent volume of vehicle, for 16h and then lysed. EPS8 was immunoprecipitated as described in Methods and proteins resolved by SDS-PAGE and western blotted with anti-phosphotyrosine antibody (A). Subsequently, antibody was removed and the blot incubated with EPS8 antibody (B). TCL, 50 µg total cell lysate (5% input). **C. & D.** HN4/FFFF cells, cultured in the presence or absence of dasatinib, were lysed, EPS8 immunoprecipitated, and western blotted with anti-phosphotyrosine (C) followed by antibody removal and reprobing with anti-EPS8 (D).

Figure S3: EPS8 Src phosphorylation site mutants show reduced exit from G2/M. The indicated cells were trypsinized, counted, fixed, stained and analyzed by flow cytometry, as described in Methods.

Figure S4: Impact of Src phosphorylation site mutants of EPS8 on keratin expression. HN4 parental cells, or cells overexpressing wild-type EPS8, FFFF-, FFF-, or 602F-EPS8, or HN12 cells as

control, were grown in triplicate on coverslips, fixed with cold methanol, and immunostained with keratin antibody (A) or with mouse IgG as a negative control (B). Samples were stained with AlexaFluor488-conjugated secondary antibodies and counterstained with DAPI. Original magnification, x400. (C). Cultures of the indicated cell lines were photographed at 100x under phase contrast. Bar = 50 µm.

Figure S5: Increased kinase activity in FFFF- and 602F-EPS8-expressing cells. Total protein lysates were prepared from cell lines, resolved by SDS-PAGE, and western blotted sequentially with the indicated antibodies.

Figure S6: Expression of PTEN in HN4 and derivative cell lines. Total protein lysates were prepared from the indicated cell lines, resolved by SDS-PAGE, and western blotted sequentially with PTEN and GAPDH antibodies.

Legends to Supplementary Tables:

Table S1. Genes showing significant downregulation in 602F-EPS8-expressing HN4 cells.

Table S2. Genes showing significant downregulation in dasatinib-treated HN4 cells.

Table S3. Genes showing significant upregulation in 602F-EPS8-expressing HN4 cells.

Table S4. Genes showing significant upregulation in dasatinib-treated HN4 cells.

Supplementary Table S1

Category	ID	Description	GeneRatio	BgRatio	pvalue	padj	geneID	Count
KEGG	hsa04110	Cell cycle	65/1070	124/5712	1.51E-17	3.25E-15	CDC27/CREBBP/DBF4/E2F2/ANAPC4/CUL1/SMC1A/MCM2/MCM6/RBL1/ORC1/ORC6/CDC45/CDC6/CDC7/MCM5/EP300/CDC25B/E2F1/STAG2/MCM4/CDK6/SMC3/YWHAE/MCM3/TTK/SKP1/ORC2/ORC4/HDAC1/CDC20/STAG1/CDKN2C/PKMYT1/PCNA/CCNB1/YWHAQ/CDK4/ESPL1/MDM2/RB1/CCNA2/SKP2/CHEK1/ANAPC1/BUB1B/CCNB2/CDC25C/CDC25A/MAD2L1/RAD21/ORC5/YWHAZ/WEE1/MCM7/PLK1/YWHAB/BUB1/CDK1/ATR/CCNE2/ANAPC7/HDAC2/TFDP1/PRKDC	65
KEGG	hsa03050	Proteasome	30/1070	43/5712	3.13E-13	3.37E-11	PSMB1/PSMC4/PSMA4/PSMC5/PSMC6/PSMA3/PSMC1/PSMB5/PSME2/PSMA7/PSMD7/PSMA2/PSMD3/PSMD11/PSMD14/PSMF1/PSMB2/PSMA1/PSME3/POMP/PSMB7/PSMB6/PSMA5/PSMD4/PSMB4/PSMC2/PSMD6/PSMC3/PSMD1/PSMD12	30
KEGG	hsa03013	RNA transport	65/1070	147/5712	5.78E-13	4.14E-11	ELAC2/TACC3/STRAP/NDC1/DDX20/NUP133/TRNT1/NUP37/THOC1/XPO1/SEH1L/NUP50/THOC5/EIF5/ACIN1/NXT2/EIF3J/POP1/POP4/EIF3B/EIF3A/NUP88/EIF4G2/MAGOHB/EIF2B1/NUP107/NUP155/EIF4G1/SUMO1/NUP43/RPP40/NUP153/UPF3B/NUP85/EIF2S2/NUP214/RAN/EIF2S1/NCBP1/TGS1/ELAC1/RPP30/UPF2/GEMIN6/RPP38/RANBP2/EIF5B/NXF1/MAGOH/NUP35/THOC7/PHAX/RPP25L/POP5/UPF3A/SNUPN/PYM1/PAIP1/POP7/EIF1AX/RPP25/ALYREF/EIF3C/XPOT/NUP62	65
KEGG	hsa03008	Ribosome biogenesis in eukaryotes	40/1070	72/5712	2.44E-12	1.31E-10	UTP18/LSG1/NOP58/RIOK2/TCOF1/XPO1/XRN2/NOP56/NXT2/POP1/POP4/FBL/RBM28/GTPBP4/MDN1/DROSHA/HEATR1/FCF1/MPHOSPH10/RPP40/GNL3L/DKC1/RAN/NAT10/NVL/RPP30/RPP38/UTP14A/NXF1/WDR43/UTP15/RPP25L/NOL6/POP5/POP7/IMP3/RPP25/RRP7A/EIF6/UTP14C	40
KEGG	hsa03030	DNA replication	26/1070	36/5712	3.47E-12	1.49E-10	POLA2/RFC2/MCM2/MCM6/POLD3/MCM5/POLE2/POLA1/MCM4/RNASEH2A/LIG1/SSBP1/RPA3/POLD2/RFC5/MCM3/RPA1/PCNA/RFC3/RNASEH2B/DNA2/PRIM2/MCM7/FEN1/POLE/PRIM1	26
KEGG	hsa03040	Spliceosome	55/1070	125/5712	4.70E-11	1.68E-09	AQR/U2AF2/THOC1/WBP11/CDC5L/HNRNPM/SNRPD3/ACIN1/PRPF6/CRNKL1/PQBP1/PRPF31/LSM5/DDX5/EFTUD2/DHX15/HSPA8/PRPF19/MAGOHB/SRSF3/SRSF6/SNRPC/SNRPB/RBM17/HNRNPA1/SRSF1/TRA2B/PRPF4/NCBP1/SNRPF/TXNL4A/SF3B4/SNRPG/DDX46/ZMAT2/RBMX/HNRNPU/CCDC12/SRSF2/MAGOH/LSM6/TRA2A/HNRNPK/SNRPD1/HNRNPA3/LSM3/PLRG1/DDX23/PUF60/SNRPE/SF3A3/ALYREF/SRSF10/SF3B3/PRPF40A	55
KEGG	hsa03440	Homologous recombination	17/1070	28/5712	1.02E-06	3.12E-05	MRE11A/RAD51/POLD3/RAD54L/NBN/SSBP1/RPA3/POLD2/RAD51C/RAD50/RPA1/BRCA2/MUS81/TOP3A/XRCC2/RAD54B/BLM	17
KEGG	hsa05016	Huntington's disease	59/1070	182/5712	5.09E-06	0.00013673	CREBBP/NDUFS1/CASP8/VDAC3/REST/POLR2E/POLR2F/EP300/CREB3/TFAM/PPARGC1A/ATP5B/COX6A1/ATP5F1/HDAC1/CREB1/NDUFB3/DNAL1/APAF1/CLTA/TAF4/NDUFA9/SDHC/CREB3L4/POLR2D/NDUFS6/NDUFB9/NDUFC2/ATP5A1/ATP5G3/ATP5J/GNAQ/DNAH3/ATP5G1/NDUFV3/CASP3/COX6C/NDUFB6/ATP5C1/ATP5H/UQCRFS1/PPID/CYCS/COX8A/POLR2L/CYC1/PLCB1/NDUFB1/NDUFA6/SP1/NDUFA4/HDAC2/COX2/CYTB/COX1/ATP6/DCTN1/ATP8/ATP5O	59
KEGG	hsa03430	Mismatch repair	14/1070	23/5712	9.10E-06	0.0002173	RFC2/MLH1/POLD3/MSH2/LIG1/SSBP1/RPA3/POLD2/RFC5/PMS2/RPA1/PCNA/RFC3/EXO1	14
KEGG	hsa05012	Parkinson's disease	45/1070	130/5712	1.04E-05	0.0002242	NDUFS1/VDAC3/ATP5B/COX6A1/PARK7/ATP5F1/NDUFB3/APAF1/NDUFA9/SDHC/NDUFS6/NDUFB9/NDUFC2/ATP5A1/ATP5G3/ATP5J/UBE2L6/ATP5G1/NDUFV3/CASP3/COX6C/NDUFB6/ATP5C1/ATP5H/UQCRFS1/UBB/GPR37/PPID/CYCS/COX8A/CYC1/NDUFB1/NDUFA6/UBE2L3/NDUFA4/COX2/CYTB/ND2/ND5/COX1/ND3/ND1/ATP6/ATP8/ATP5O	45
KEGG	hsa00190	Oxidative phosphorylation	45/1070	132/5712	1.63E-05	0.00031902	NDUFS1/ATP5B/COX6A1/ATP6V1B1/ATP5F1/ATP6V0B/NDUFB3/ATP6V1G1/NDUFA9/SDHC/NDUFS6/NDUFB9/NDUFC2/ATP5A1/ATP5G3/ATP5J/ATP6V1C1/ATP5G1/ATP6V0D1/NDUFV3/COX6C/NDUFB6/ATP5C1/COX11/ATP5L/ATP5H/UQCRFS1/ATP6V0E2/COX8A/CYC1/NDUFB1/NDUFA6/NDUFA4/COX2/CYTB/ND2/ND5/COX1/ND3/ND1/ATP6/ATP8/ATP5J2/ATP5O/ATP6V1E2	45
KEGG	hsa00310	Lysine degradation	20/1070	44/5712	4.27E-05	0.00076573	ACAT1/SETD1A/SUV39H1/GCDH/OGDH/AADAT/WHSC1/EHHADH/ASH1L/ACAT2/ECHS1/SETD1B/SETD7/SUV39H2/NSD1/SETMAR/EHMT1/SETD2/KMT5A/EHMT2	20
KEGG	hsa00020	Citrate cycle (TCA cycle)	15/1070	30/5712	0.00010226	0.00169121	MDH1/DLD/FH/ACO2/PCK2/OGDH/ACLY/PDHA1/SUCLA2/IDH1/SDHC/DLAT/SUCLG1/IDH3A/PDHB	15
KEGG	hsa00240	Pyrimidine metabolism	34/1070	99/5712	0.00014782	0.00227007	POLR3B/POLA2/RRM2B/POLR1A/NT5C2/POLD3/POLR2E/POLR2F/POLR3H/POLE2/POLA1/POLD2/UMPS/ITPA/DUT/UCK1/CMPK2/PNPT1/POLR2D/PRIM2/POLR3K/RRM1/TK1/DTYMK/CTPS1/RRM2/NME6/TYMS/POLE/POLR2L/POLR3C/ENTPD5/PRIM1/NME1	34
KEGG	hsa04120	Ubiquitin mediated proteolysis	42/1070	134/5712	0.00026819	0.0038441	CDC27/BRCA1/BIRC3/PIAS1/CUL3/NEDD4L/ANAPC4/CUL1/UBE2K/KEAP1/HUWE1/PPIL2/MID1/CUL2/UBE2S/PRPF19/BIRC2/SKP1/FANCL/CDC20/UBA2/HERC2/UBE4B/MDM2/CUL4A/SAE1/RFWD2/SKP2/UBE3B/FBXO4/ANAPC1/TCEB1/UBE2L6/UBE2Q1/UBE2E1/UBE2C/UBE2N/UBE2L3/NHLRC1/ANAPC7/WWP2/KLHL9	42
KEGG	hsa03410	Base excision repair	15/1070	33/5712	0.00038907	0.00522812	POLB/UNG/POLD3/POLE2/APEX1/PARP4/LIG1/POLD2/NEIL3/SMUG1/PCNA/PARP1/FEN1/POLE/HMGB1	15
KEGG	hsa03450	Non-homologous end-joining	8/1070	13/5712	0.00077676	0.00982376	MRE11A/XRCC5/RAD50/XRCC4/DCLRE1C/FEN1/XRCC6/PRKDC	8
KEGG	hsa03420	Nucleotide excision repair	17/1070	43/5712	0.00116085	0.01386568	RFC2/POLD3/POLE2/LIG1/RPA3/POLD2/GTF2H1/GTF2H3/RFC5/RAD23B/RPA1/PCNA/RFC3/CUL4A/CETN2/XPC/POLE	17
KEGG	hsa05010	Alzheimer's disease	47/1070	166/5712	0.00148292	0.01678041	NDUFS1/CASP8/HSD17B10/MAPK1/ATP5B/COX6A1/ATP5F1/APH1A/NDUFB3/IDE/APAF1/ITPR2/ADAM10/NDUFA9/SDHC/CALM2/NDUFS6/NDUFB9/NDUFC2/ATP5A1/ATP5G3/ATP5J/GNAQ/ATP5G1/NDUFV3/CASP3/COX6C/NDUFB6/ATP5C1/ATP5H/FADD/UQCRFS1/CYCS/COX8A/CYC1/PLCB1/NDUFB1/NDUFA6/NDUFA4/CALM1/COX2/CYTB/COX1/ATP6/PSENEN/ATP8/ATP5O	47
KEGG	hsa04114	Oocyte meiosis	34/1070	112/5712	0.00184795	0.01986543	CDC27/ANAPC4/CUL1/RPS6KA6/SMC1A/AURKA/MAPK1/SMC3/YWHAE/FBXO5/SKP1/PPP2CA/CDC20/ITPR2/PKMYT1/SGO1/CCNB1/YWHAQ/ESPL1/PPP2R1B/ADCY3/CALM2/ANAPC1/CCNB2/CDC25C/MAD2L1/YWHAZ/PLK1/YWHAB/BUB1/CDK1/CCNE2/ANAPC7/CALM1	34
KEGG	hsa05215	Prostate cancer	27/1070	87/5712	0.00372977	0.03818574	CREBBP/E2F2/FGFR2/HSP90AA1/MAPK1/EP300/NFKBIA/E2F1/PIK3CG/CREB3/NFKB1/PIK3R3/CREB1/ATF4/RAF1/MDM2/RB1/PDPK1/ERBB2/CREB3L4/PIK3R1/EGFR/TCF7L2/NKX3-1/HRAS/CCNE2/MTOR	27

Supplementary Table S2

Category	ID	Description	GeneRatio	BgRatio	pvalue	padj	geneID	Count
KEGG	hsa03030	DNA replication	30/837	36/5712	4.87E-20	1.02E-17	POLA2/RFC1/RFC2/POLD1/MCM2/MCM6/POLD3/MCM5/POLE2/POLA1/MCM4/RNASEH2A/LIG1/RPA3/POLD2/RFC5/MCM3/RPA2/RPA1/PCNA/RFC3/RNASEH2B/DNA2/PRIM2/POLE3/RFC4/MCM7/FEN1/POLE/PRIM1	30
KEGG	hsa04110	Cell cycle	60/837	124/5712	1.77E-19	1.86E-17	CDC27/DBF4/E2F2/SMC1A/MCM2/MCM6/RBL1/CDC14B/ORC1/ORC6/CDC45/CDC6/CDC7/MCM5/CDC25B/E2F1/STAG2/MCM4/SMC3/YWHAE/CCND1/CDKN1B/MCM3/CCND3/TTK/CDC20/CCND2/CDKN2C/CDK2/PKMYT1/ANAPC13/PCNA/CCNB1/ORC3/CDK4/ESPL1/ANAPC11/CCNA2/SKP2/CHEK1/ANAPC1/BUB3/BUB1B/CCNB2/CDC25C/CDC25A/MAD2L1/PTTG1/RAD21/WEE1/MCM7/PLK1/BUB1/CDK1/ATR/CCNE2/CDC26/CHEK2/TFDP1/PRKDC	60
KEGG	hsa03040	Spliceosome	56/837	125/5712	2.48E-16	1.74E-14	AQR/SNRNP40/U2AF2/SNRPA/WBP11/CHERP/SF3B2/HNRNPC/HNRNPM/SF3A1/SNRPD3/PHF5A/PRPF6/PQBP1/LSM5/HSPA8/PRPF19/MAGOHB/SRSF3/TCERG1/SF3B6/SMNDC1/SNRPC/SNRPB/LSM7/LSM4/SNRPA1/PRPF38A/HNRNPA1/SRSF1/TRA2B/PRPF4/NCBP1/PPIL1/SNRPF/DHX38/SNRPG/RBMX/HNRNPU/CCDC12/SRSF2/MAGOH/U2SURP/LSM6/HNRNPK/SNRPD1/HNRNPA3/LSM3/PPIH/PRPF8/PUF60/SNRPE/SF3A3/ALYREF/SF3B3/LSM2	56
KEGG	hsa03050	Proteasome	26/837	43/5712	5.05E-12	2.65E-10	PSMB1/PSMC4/PSMA4/PSME1/PSMD8/PSMC6/PSMA3/PSMC1/PSMB5/PSME2/PSMA7/PSMD7/PSMD3/PSMD11/PSMD14/PSMB2/POMP/PSMB7/PSMB6/PSMA5/PSMC2/PSMD6/PSMC3/PSMD1/PSMB8/PSMB9	26
KEGG	hsa03430	Mismatch repair	17/837	23/5712	2.42E-10	1.02E-08	RFC1/RFC2/POLD1/MLH1/POLD3/MSH2/LIG1/RPA3/POLD2/RFC5/MSH6/RPA2/RPA1/PCNA/RFC3/RFC4/EXO1	17
KEGG	hsa03013	RNA transport	50/837	147/5712	2.04E-09	7.15E-08	TACC3/STRAP/NDC1/PABPC1/NUP37/XPO1/SEH1L/PABPC4/GEMIN2/NUP50/AAAS/NUP188/RANGAP1/PRMT5/NXT2/NUP93/EIF3J/EIF3E/EIF3A/KPNB1/NUP88/NUP98/MAGOHB/NUP107/NUP155/EIF4G1/NUP43/NUP153/UPF3B/NUP85/EIF2S2/NUP214/THOC6/NUP210/RAN/NCBP1/NUP58/EIF3H/RPP30/UPF2/NUP205/SEC13/MAGOH/NUP35/THOC7/RPP25L/EIF1AX/RPP25/ALYREF/XPOT	50
KEGG	hsa03410	Base excision repair	18/837	33/5712	9.60E-08	2.72E-06	LIG3/POLD1/NTHL1/XRCC1/UNG/POLD3/POLE2/APEX1/LIG1/POLD2/NEIL3/PCNA/MUTYH/PARP1/POLE3/FEN1/POLE/HMGB1	18
KEGG	hsa00020	Citrate cycle (TCA cycle)	17/837	30/5712	1.04E-07	2.72E-06	MDH1/CS/SDHA/FH/ACO2/PCK2/SDHB/ACO1/ACLY/PDHA1/SUCLA2/IDH1/MDH2/DLAT/SUCLG1/IDH3A/SDHD	17
KEGG	hsa03440	Homologous recombination	16/837	28/5712	2.12E-07	4.95E-06	RAD51/POLD1/POLD3/RAD54L/NBN/RPA3/POLD2/RAD51C/RPA2/RPA1/BRCA2/EME1/TOP3A/XRCC2/RAD54B/BLM	16
KEGG	hsa03420	Nucleotide excision repair	20/837	43/5712	5.65E-07	1.19E-05	RFC1/ERCC8/RFC2/POLD1/POLD3/POLE2/LIG1/RPA3/POLD2/RFC5/RPA2/RAD23B/RPA1/PCNA/RFC3/POLE3/XPC/RFC4/POLE/RAD23A	20
KEGG	hsa05012	Parkinson's disease	40/837	130/5712	1.72E-06	3.29E-05	SLC25A5/NDUFS1/SDHA/VDAC3/NDUFC1/ATP5B/COX6A1/COX7A2/PARK7/ATP5F1/SDHB/NDUFA8/APAF1/COX6B1/COX7B/NDUFB5/NDUFA9/NDUFS6/NDUFB9/ATP5A1/ATP5G3/ATP5J/UBE2L6/ATP5G1/COX6C/NDUFB6/ATP5C1/UQCRFS1/UBB/CYCS/UQCRH/COX8A/COX5A/CYC1/NDUFB1/UQCR10/NDUFA12/NDUFA6/UBE2J1/SDHD	40
KEGG	hsa00240	Pyrimidine metabolism	31/837	99/5712	1.71E-05	0.00028614	POLA2/POLD1/POLD3/CAD/POLR2E/UPB1/POLE2/POLA1/POLD2/UMPS/NT5C3A/DUT/POLR1E/ENTPD1/POLR2D/PRIM2/POLE3/DCK/POLR3K/RRM1/TK1/POLR2G/DTYMK/CTPS1/RRM2/TYMS/POLE/POLR2L/PRIM1/TXNRD1/NME1	31
KEGG	hsa00190	Oxidative phosphorylation	38/837	132/5712	1.77E-05	0.00028614	COX10/COX15/NDUFS1/SDHA/NDUFC1/ATP5B/COX6A1/COX7A2/ATP5F1/SDHB/NDUFA8/COX6B1/COX7B/NDUFB5/NDUFA9/NDUFS6/NDUFB9/ATP5A1/ATP5G3/ATP5J/ATP5G1/COX6C/NDUFB6/ATP5C1/ATP5L/UQCRFS1/ATP6V0E2/UQCRH/COX8A/COX5A/CYC1/PPA1/NDUFB1/UQCR10/NDUFA12/NDUFA6/SDHD/ATP6V1E2	38
KEGG	hsa01100	Metabolic pathways	207/837	1127/5712	7.11E-05	0.00106639	LAP3/COX10/AASS/REV3L/POLA2/MDH1/COX15/NDUFS1/ADSS/MAT2B/DGKG/ALDH18A1/BCAT1/CS/POLD1/ME1/MTHFD2/COASY/ST6GALNAC1/ASNS/MGAT4A/RDH11/ALDH3A2/SDHA/POLD3/MCCC1/FDFT1/ARG2/CAD/SEPHS1/DNMT3B/FH/PHGDH/PTGS1/NANS/POLR2E/GGT5/UPB1/PRODH/PNPLA3/ACO2/POLE2/ITPK1/MTHFD1/PCK2/AHCY/PIGU/POLA1/PRPS2/IL4I1/ISYNA1/POLD2/ALDH3A1/NDUFC1/GALNT7/PDHX/CHKA/ATP5B/LDHB/COX6A1/GMDS/PCCB/UMPS/ODC1/DHCR24/ATP5F1/SRM/CTH/AGMAT/ST6GALNAC5/SDHB/AKR1A1/PRDX6/NDUFA8/ALDH6A1/GOT1/ACAT2/NT5C3A/ACO1/ACSL3/PTGIS/GOT2/COX6B1/PAICS/PPAT/MGAT3/DUT/ASS1/DNMT1/COX7B/PPT1/ACLY/PDHA1/GCH1/HSD17B7/STT3A/PSAT1/SUCLA2/NDUFB5/EPRS/SMPD4/ST6GALNAC4/POLR1E/SELENOI/ATIC/IDH1/PPCDC/GPAT3/ALG10/NDUFA9/MMAB/GCNT3/PGD/PIGK/ALDH9A1/POLR2D/NDUFS6/PRIM2/MDH2/PSPH/EBP/NDUFB9/UGCG/POLE3/PTGES/GLUD1/HSD17B12/APIP/DLAT/ATP5A1/RPIA/DGKE/ATP5G3/ATP5J/ACSS1/ADK/DCK/GALNT14/GART/ATP5G1/G6PD/FDPS/BDH1/ALOX15/POLR3K/PLPP3/STT3B/SUCLG1/GMPS/TKT/CMBL/ALDH7A1/COX6C/NDUFB6/PIGO/ATP5C1/GPT2/CKB/IDH3A/ATP5L/RRM1/TK1/POLR2G/DTYMK/AHCYL1/UQCRFS1/B3GALNT1/FASN/DCXR/ATP6V0E2/CTPS1/RRM2/QARS/MBOAT1/DPAGT1/LCLAT1/HPSE/COQ2/UQCRH/ALG10B/COX8A/TYMS/SHMT1/POLE/POLR2L/IMPDH2/GLDC/COX5A/PFAS/CYC1/SHMT2/ADI1/PYCR1/NDUFB1/UQCR10/PGP/NDUFA6/PCYT2/PRIM1/AKR1B10/SGMS1/SDHD/ALG3/DHFR/NME1/ATP6V1E2/HMBS	207
KEGG	hsa05016	Huntington's disease	46/837	182/5712	9.14E-05	0.00128023	SLC25A5/NDUFS1/SDHA/VDAC3/POLR2E/TFAM/NDUFC1/PPARGC1A/ATP5B/COX6A1/COX7A2/IFT57/ATP5F1/SDHB/NDUFA8/APAF1/CLTA/COX6B1/COX7B/NDUFB5/NDUFA9/POLR2D/NDUFS6/NDUFB9/ATP5A1/ATP5G3/ATP5J/GNAQ/ATP5G1/COX6C/NDUFB6/ATP5C1/POLR2G/UQCRFS1/CYCS/UQCRH/COX8A/POLR2L/COX5A/CYC1/NDUFB1/UQCR10/NDUFA12/NDUFA6/SP1/SDHD	46
KEGG	hsa00970	Aminoacyl-tRNA biosynthesis	16/837	41/5712	0.00010993	0.00144287	LARS2/KARS/AARS/GARS/TARS/FARSB/DARS2/LARS/NARS/YARS/EPRS/WARS/MARS/QARS/IARS/VARS	16
KEGG	hsa03008	Ribosome biogenesis in euka	23/837	72/5712	0.00014774	0.00182505	UTP18/NOP58/TCOF1/XPO1/NOP56/NXT2/FBL/RBM28/MDN1/DROSHA/FCF1/RCL1/RIOK1/GNL3L/DKC1/RAN/IMP4/NHP2/RPP30/GNL3/RPP25L/NOL6/RPP25	23
KEGG	hsa00270	Cysteine and methionine met	14/837	36/5712	0.00030773	0.00359017	MAT2B/DNMT3B/AHCY/IL4I1/LDHB/SRM/CTH/GOT1/GOT2/DNMT1/ENOPH1/APIP/AHCYL1/ADI1	14
KEGG	hsa04114	Oocyte meiosis	30/837	112/5712	0.00053119	0.0058711	CDC27/PPP2R5A/SMC1A/AURKA/SMC3/YWHAE/FBXO5/CDC20/CDK2/PKMYT1/ANAPC13/SGO1/CCNB1/ESPL1/PPP2R1B/ADCY3/ANAPC11/CALM2/ANAPC1/PPP2R5E/CCNB2/CDC25C/MAD2L1/PTTG1/PLK1/BUB1/CDK1/CCNE2/CDC26/PPP1CC	30
KEGG	hsa00620	Pyruvate metabolism	14/837	39/5712	0.00079782	0.00808851	MDH1/ME1/ALDH3A2/ME2/PCK2/LDHB/ACAT2/GLO1/PDHA1/ALDH9A1/MDH2/DLAT/ACSS1/ALDH7A1	14
KEGG	hsa03015	mRNA surveillance pathway	23/837	80/5712	0.00080885	0.00808851	PPP2R5A/PABPC1/DAZAP1/PAPOLA/PABPC4/CSTF2/NXT2/MAGOHB/CPSF6/SMG7/CPSF3/UPF3B/MSI1/NCBP1/PPP2R1B/FIP1L1/UPF2/PPP2R5E/MAGOH/CPSF2/NUDT21/CSTF3/ALYREF	23
KEGG	hsa04115	p53 signaling pathway	20/837	68/5712	0.00127776	0.01219679	BID/GTSE1/TP73/CCND1/CCND3/CCNG1/CCND2/APAF1/CDK2/CCNB1/CDK4/PMAIP1/CHEK1/CCNB2/CDK1/RRM2/CYCS/ATR/CCNE2/CHEK2	20
KEGG	hsa00630	Glyoxylate and dicarboxylate	8/837	18/5712	0.00229424	0.01950479	MDH1/CS/ACO2/PCCB/ACAT2/ACO1/MDH2/PGP	8
KEGG	hsa00670	One carbon pool by folate	8/837	18/5712	0.00229424	0.01950479	MTHFD2/MTHFD1/ATIC/GART/TYMS/SHMT1/SHMT2/DHFR	8
KEGG	hsa00290	Valine, leucine and isoleucine	6/837	Nov-12	0.002322	0.01950479	LARS2/BCAT1/PDHA1/LARS/IARS/VARS	6
KEGG	hsa05010	Alzheimer's disease	38/837	166/5712	0.00268308	0.02167106	BID/NDUFS1/SDHA/NDUFC1/ATP5B/COX6A1/COX7A2/ATP5F1/SDHB/NDUFA8/IDE/APAF1/COX6B1/COX7B/NDUFB5/NDUFA9/CALM2/NDUFS6/NDUFB9/ATP5A1/ATP5G3/ATP5J/GNAQ/ATP5G1/COX6C/NDUFB6/ATP5C1/UQCRFS1/CYCS/UQCRH/COX8A/COX5A/CYC1/NDUFB1/UQCR10/NDUFA12/NDUFA6/SDHD	38
KEGG	hsa00330	Arginine and proline metabol	16/837	54/5712	0.0035003	0.02722457	LAP3/ALDH18A1/ALDH3A2/ARG2/PRODH/ODC1/SRM/AGMAT/GOT1/GOT2/ASS1/ALDH9A1/GLUD1/ALDH7A1/CKB/PYCR1	16
KEGG	hsa03018	RNA degradation	19/837	70/5712	0.00456053	0.03420398	EDC4/SKIV2L2/PABPC1/PABPC4/LSM5/EXOSC3/HSPA9/EXOSC8/EXOSC9/LSM7/LSM4/EXOSC2/TOB1/HSPD1/CNOT9/LSM6/LSM3/DCP2/LSM2	19
KEGG	hsa04914	Progesterone-mediated oocy	22/837	86/5712	0.00519254	0.03760119	CDC27/HSP90AA1/HSP90AB1/CDC25B/PIK3CG/PIK3R3/CDK2/PKMYT1/ANAPC13/CCNB1/ADCY3/ANAPC11/CCNA2/ANAPC1/CCNB2/CDC25C/CDC25A/MAD2L1/PLK1/BUB1/CDK1/CDC26	22
KEGG	hsa00230	Purine metabolism	36/837	162/5712	0.00572702	0.04008917	POLA2/ADSS/GUCY1B3/POLD1/POLD3/POLR2E/POLE2/GMPR2/POLA1/PRPS2/POLD2/NT5C3A/PAICS/PPAT/POLR1E/ADCY3/ENTPD1/ATIC/POLR2D/PRIM2/POLE3/ADK/DCK/GART/POLR3K/GMPS/NUDT5/RRM1/POLR2G/RRM2/POLE/POLR2L/IMPDH2/PFAS/PRIM1/NME1	36
KEGG	hsa03450	Non-homologous end-joining	6/837	13/5712	0.00663692	0.04495978	XRCC5/XRCC4/DCLRE1C/FEN1/XRCC6/PRKDC	6

Supplementary Table S3

Category	ID	Description	GeneRatio	BgRatio	pvalue	padj	geneID
KEGG	hsa03010	Ribosome	44/994	88/5712	1.71E-12	2.00E-10	RPL18/RPL31/RPS5/RPLP0/RPL3/RPS19/RPL18A/RPL28/RPL34/RPS13/RPS12/RPL24/RPS25/RPL21/RPL23/RPL36/RPL27/RPLP1/RPS24/RPL3L/RPS11/RPL13A/RPL11/RPS8/RPL32/RPL37/RPL10/RPL7A/RPS3/RPL29/RPL22L1/RPS14/RPL27A/RPL13/RPS21/RPLP2/RPL37A/RPL12/RPS4X/RPL10A/RPL39/UBA52/RPS18/RPS28
KEGG	hsa04512	ECM-receptor interaction	43/994	85/5712	1.84E-12	2.00E-10	ITGA3/ITGA2B/CD44/TNC/LAMC2/ITGB5/LAMB1/LAMA1/COMP/COL1A1/LAMA4/THBS4/ITGB6/FN1/SDC4/COL5A1/ITGB4/COL4A2/CD36/ITGA7/LAMC1/CHAD/ITGA11/ITGAV/COL6A1/COL6A2/HSPG2/ITGB1/ITGA5/SDC3/ITGA2/TNXB/LAMB2/GP1BA/THBS2/COL4A1/COL4A5/AGRN/CD47/LAMB3/COL4A6/COL5A2/ITGB3
KEGG	hsa04510	Focal adhesion	71/994	200/5712	3.05E-10	2.21E-08	ITGA3/ITGA2B/FYN/VCL/TNC/PIK3CB/LAMC2/MYLK/ACTN1/ITGB5/PXN/LAMB1/PDGFB/MYL9/MYL12A/LAMA1/COMP/MYL7/COL1A1/VEGFA/LAMA4/THBS4/PDGFRB/ITGB6/FN1/AKT3/MYL12B/CCND2/PGF/PRKCG/RAC2/FLNC/SHC2/COL5A1/ITGB4/COL4A2/ITGA7/LAMC1/FLNB/CHAD/ITGA11/ITGAV/PARVG/IGF1R/COL6A1/COL6A2/PAK1/ITGB1/BRAF/ZYX/VAV2/SHC1/ITGA5/ITGA2/TNXB/PIK3CD/LAMB2/VEGFB/JUN/ACTG1/THBS2/COL4A1/COL4A5/PARVB/LAMB3/FLNA/SRC/PDGFA/COL4A6/COL5A2/ITGB3
KEGG	hsa04514	Cell adhesion molecules (CAMs)	45/994	130/5712	1.34E-06	7.26E-05	CD99/CD4/CD6/CNTN1/CDH3/NEO1/PVR/SIGLEC1/GLG1/NRCAM/MADCAM1/CD40/CD276/CLDN15/NRXN2/CD274/SDC4/CDH15/ITGAV/PTPRF/ESAM/ITGB1/F11R/CLDN14/SDC3/NFASC/CLDN19/JAM3/ITGAM/NLGN2/CDH2/PTPRM/HLA-DQB1/HLA-DRB1/NLGN3/HLA-DQA1/PDCD1LG2/L1CAM/HLA-C/HLA-E/HLA-F/HLA-A/CLDN9/HLA-B/HLA-DOB
KEGG	hsa04142	Lysosome	41/994	121/5712	7.26E-06	0.00031507	LAMP2/HEXB/GALC/AP1M1/GGA1/ARSA/LGMN/CTSZ/ACP5/PLA2G15/MAN2B1/DNASE2/ATP6V0A4/AP1S1/ABCA2/MANBA/TCIRG1/CTSD/NPC2/IDUA/NAPSA/CD63/SCARB2/GALNS/AP3S2/LAPTM5/CTSB/SMPD1/TPP1/GUSB/GLB1/GAA/CTSF/CLTB/AP3S1/FUCA1/SGSH/GM2A/PSAP/NEU1/CTSO
KEGG	hsa05410	Hypertrophic cardiomyopathy (HCM)	30/994	83/5712	3.04E-05	0.00109959	ITGA3/ITGA2B/CACNA2D2/ITGB5/CACNA1F/TGFB1/CACNG7/ITGB6/TNNT2/TNNI3/CACNG6/PRKAB2/ITGB4/ITGA7/ITGA11/ITGAV/TPM1/CACNG8/ITGB1/ACE/ITGA5/SGCB/ITGA2/TPM4/CACNB3/ATP2A2/ACTG1/DMD/TNF/ITGB3
KEGG	hsa04141	Protein processing in endoplasmic reticulum	48/994	165/5712	0.00011655	0.0033912	HSPA5/HERPUD1/ERLEC1/SEL1L/LMAN1/SEC31B/MAP2K7/ERO1B/PPP1R15A/EDEM2/GANAB/DNAJB11/SEC23A/UGGT2/WFS1/MAN1A1/SEC24A/SSR3/MOGS/FBXO2/MAN1C1/HSPA2/DAD1/PRKCSH/EDEM1/OS9/DNAJB2/SEC31A/PDIA6/HYOU1/SEC24D/PDIA4/SEC13/SYVN1/RPN1/HSP90B1/PDIA3/LMAN2/EIF2AK3/DDIT3/SEC24C/MAN1B1/CALR/SSR4/P4HB/ERO1A/RNF5/DDOST
KEGG	hsa04010	MAPK signaling pathway	70/994	266/5712	0.00012502	0.0033912	CACNA1G/CACNA2D2/RASGRP2/MAP2K7/NFKB2/MAP3K4/NLK/GADD45B/MKNK2/PDGFB/NFATC4/NFATC2/CACNA1F/RELB/TGFB1/CACNG7/DUSP3/DUSP16/SRF/DUSP22/FGF1/PDGFRB/GADD45A/AKT3/DUSP1/PPP3CC/NR4A1/IL1B/RRAS/PRKCG/HSPA2/RAC2/FLNC/ECSIT/CACNG6/DUSP9/NGF/MAP3K7/FLNB/MYC/FGF5/MAPK8IP3/DUSP6/MAP3K12/TP53/CACNG8/RASA1/PAK1/RASA2/MAPK13/BRAF/MRAS/PLA2G2F/TAOK1/FGF11/DUSP7/CACNB3/MAP4K2/JMJD7-PLA2G4B/RASGRP4/RELA/DDIT3/JUN/DUSP8/MAPT/CACNA1H/FLNA/PDGFA/NTRK1/TNF
KEGG	hsa05414	Dilated cardiomyopathy	30/994	90/5712	0.00016932	0.00408251	ITGA3/ITGA2B/CACNA2D2/ITGB5/CACNA1F/TGFB1/CACNG7/ITGB6/TNNT2/ADCY4/TNNI3/CACNG6/ITGB4/ITGA7/ITGA11/ITGAV/TPM1/CACNG8/ITGB1/ITGA5/SGCB/ITGA2/TPM4/CACNB3/ADCY6/ATP2A2/ACTG1/DMD/TNF/ITGB3
KEGG	hsa05222	Small cell lung cancer	28/994	84/5712	0.00027666	0.00600361	ITGA3/ITGA2B/PIK3CB/LAMC2/PTGS2/PIAS2/TRAF5/LAMB1/LAMA1/LAMA4/FN1/AKT3/PIAS3/COL4A2/LAMC1/MYC/ITGAV/TP53/ITGB1/ITGA2/PIK3CD/LAMB2/RELA/COL4A1/COL4A5/LAMB3/COL4A6/RXRB
KEGG	hsa05200	Pathways in cancer	80/994	323/5712	0.00036145	0.00713038	ITGA3/ITGA2B/DAPK2/PIK3CB/LAMC2/RASSF1/PTGS2/NFKB2/PIAS2/TRAF5/MMP2/LAMB1/PDGFB/HIF1A/MMP9/LAMA1/AXIN1/TGFB1/DVL1/CBL/WNT5B/PPARD/VEGFA/LAMA4/FGF1/PDGFRB/FN1/AKT3/SLC2A1/PGF/PLCG1/CDKN1A/PAX8/PRKCG/RAC2/PIAS3/WNT2B/COL4A2/ETS1/LAMC1/MYC/ITGAV/FGF5/LEF1/IGF1R/TP53/SMAD4/CBLC/WNT9A/ITGB1/WNT7A/BRAF/FGF11/WNT4/TGFA/ITGA2/SHH/RET/HSP90B1/SMAD3/DAPK3/CXCL8/PIK3CD/LAMB2/ARNT2/RELA/VEGFB/STAT5B/FZD4/JUN/CSF1R/COL4A1/WNT7B/COL4A5/LAMB3/PDGFA/COL4A6/PLCG2/NTRK1/RXRB
KEGG	hsa05412	Arrhythmogenic right ventricular cardiomyopathy (ARVC)	25/994	74/5712	0.00046048	0.00832697	ITGA3/ITGA2B/CACNA2D2/ACTN1/ITGB5/CACNA1F/CACNG7/ITGB6/CACNG6/ITGB4/ITGA7/ITGA11/ITGAV/LEF1/CACNG8/ITGB1/ITGA5/SGCB/ITGA2/CACNB3/CDH2/ATP2A2/ACTG1/DMD/ITGB3
KEGG	hsa04670	Leukocyte transendothelial migration	34/994	114/5712	0.00068711	0.01146947	CD99/VCL/PIK3CB/ACTN1/RAPGEF3/MMP2/PXN/MMP9/MYL9/MYL12A/CLDN15/MYL7/NCF2/MYL12B/PTK2B/PLCG1/PRKCG/GNAI1/RAC2/MSN/ESAM/ITGB1/MAPK13/F11R/CLDN14/VAV2/CLDN19/JAM3/ITGAM/PIK3CD/ACTG1/PLCG2/SIPA1/CLDN9
KEGG	hsa00511	Other glycan degradation	9/994	17/5712	0.000902	0.01398094	MAN2B2/HEXB/MAN2B1/MANBA/MAN2C1/ENGASE/GLB1/FUCA1/NEU1
KEGG	hsa05146	Amoebiasis	31/994	104/5712	0.00117767	0.01703695	VCL/PIK3CB/LAMC2/ACTN1/LAMB1/PLCB4/LAMA1/TGFB1/COL1A1/LAMA4/C9/FN1/ARG1/IL1B/PRKCG/COL5A1/COL4A2/LAMC1/CSF2/CXCL8/ITGAM/PIK3CD/LAMB2/RELA/COL4A1/COL4A5/LAMB3/COL4A6/COL5A2/TNF/SERPINB10
KEGG	hsa04144	Endocytosis	51/994	201/5712	0.00240525	0.03262122	EHD3/PSD/PRKCZ/EPN2/ACAP1/ITCH/SH3GLB1/ARFGAP1/SMAD7/EEA1/TGFB1/DNM1/GIT1/FOLR3/CBL/STAMBP/HSPA2/PLD2/RAB11FIP4/ACAP3/RAB11FIP5/IGF1R/SH3GL3/MVB12A/CBLC/IQSEC1/SH3KBP1/IL2RG/PRKCI/RET/SMAD3/PIP5KL1/RAB31/GRK2/CLTB/VPS37D/CSF1R/AP2A2/ARAP1/AP2A1/SRC/DNM3/NTRK1/SMURF1/GRK5/HLA-C/HLA-E/HLA-F/HLA-A/HLA-B/ARFGAP3
KEGG	hsa04940	Type I diabetes mellitus	15/994	41/5712	0.00256728	0.03277063	CPE/IL1B/GAD1/PTPRN2/HLA-DQB1/HLA-DRB1/HLA-DQA1/HLA-C/HLA-E/HLA-F/HLA-A/LTA/TNF/HLA-B/HLA-DOB

Supplementary Table S4

Category	ID	Description	GeneRatio	BgRatio	pvalue	padj	geneID	Count
KEGG	hsa04512	ECM-receptor interaction	39/646	85/5712	6.70E-16	1.36E-13	CD44/TNC/LAMA3/LAMC2/COL5A3/ITGB5/GP6/ITGB8/COL1A1/ITGB6/FN1/SDC1/SDC4/COL5A1/ITGB4/COL4A2/ITGA7/LAMC1/ITGAV/COL6A1/COL6A2/HSPG2/ITGB1/ITGA5/SDC3/ITGA2/COL3A1/THBS3/SDC2/LAMB2/THBS2/COL4A1/COL4A5/AGRN/CD47/LAMB3/COL4A6/COL5A2/ITGB3	39
KEGG	hsa04142	Lysosome	45/646	121/5712	5.28E-14	5.36E-12	LAMP2/AGA/CTNS/GALC/CTSA/CLTCL1/GNPTG/MCOLN1/GGA1/ARSA/LGMN/CTSZ/ACP5/PLA2G15/MAN2B1/AP1S1/ABCA2/NAGLU/MANBA/CTSC/TCIRG1/GNPTAB/HYAL1/NPC2/IDUA/CD63/SCARB2/GALNS/SUMF1/LAPTM5/CTSB/HGSNAT/TPP1/GUSB/GLB1/GAA/CTSF/CLTB/LAMP1/GM2A/IGF2R/PSAP/HEXA/AP4M1/CTSO	45
KEGG	hsa04510	Focal adhesion	57/646	200/5712	9.83E-12	6.65E-10	VCL/TNC/LAMA3/LAMC2/MYLK/ACTN1/COL5A3/ITGB5/PXN/PDGFB/MYL9/MYL12A/ITGB8/MYL7/COL1A1/VEGFA/ITGB6/FN1/MYL12B/PGF/RAC2/SHC2/COL5A1/ITGB4/COL4A2/ITGA7/LAMC1/FLNB/ITGAV/PARVG/IGF1R/COL6A1/COL6A2/SHC3/ITGB1/PRKCA/ZYX/VAV2/ITGA5/ITGA2/CTNNB1/COL3A1/THBS3/PIK3CD/LAMB2/JUN/ACTG1/THBS2/COL4A1/COL4A5/LAMB3/FLNA/SRC/PDGFA/COL4A6/COL5A2/ITGB3	57
KEGG	hsa04670	Leukocyte transendothelial migration	30/646	114/5712	5.68E-06	0.00028841	CD99/VCL/CTNNA1/CYBA/ACTN1/MMP2/PXN/NCF4/MMP9/MYL9/MYL12A/MYL7/GNAI2/NCF2/MYL12B/PTK2B/PLCG1/RAC2/MSN/ITGB1/PRKCA/MAPK13/CLDN14/VAV2/CTNNB1/ITGAM/PIK3CD/ACTG1/MAPK11/PLCG2	30
KEGG	hsa05146	Amoebiasis	27/646	104/5712	2.18E-05	0.00088381	SERPINB1/VCL/LAMA3/LAMC2/GNA15/ACTN1/COL5A3/TGFB1/HSPB1/COL1A1/C9/FN1/COL5A1/COL4A2/LAMC1/PRKCA/CSF2/COL3A1/CXCL8/ITGAM/PIK3CD/LAMB2/COL4A1/COL4A5/LAMB3/COL4A6/COL5A2	27
KEGG	hsa04810	Regulation of actin cytoskeleton	44/646	210/5712	2.82E-05	0.00095463	VCL/MYLK/ACTN1/ARHGEF1/ITGB5/PXN/SLC9A1/PDGFB/MYH9/MYL9/MYL12A/ITGB8/MYL7/LIMK1/FGF1/ITGB6/FN1/MYL12B/RRAS/RAC2/ITGB4/ITGA7/ARHGEF4/ITGAV/FGF5/ITGAX/TIAM2/GNA12/MSN/GSN/ITGB1/MRAS/VAV2/ITGA5/FGF11/ITGA2/PIP4K2C/ITGAM/PIK3CD/LIMK2/ACTG1/PDGFA/BRK1/ITGB3	44
KEGG	hsa04145	Phagosome	33/646	146/5712	6.07E-05	0.00163119	LAMP2/MRC2/CYBA/SCARB1/ITGB5/NCF4/TCIRG1/NCF2/STX12/C3/TUBA4A/DYNC1LI2/TUBB2A/ITGAV/ITGB1/PLA2R1/C1R/ITGA5/ITGA2/TUBA1A/THBS3/ITGAM/TUBB6/ACTG1/HGS/LAMP1/THBS2/HLA-C/HLA-E/HLA-F/HLA-A/HLA-B/ITGB3	33
KEGG	hsa04360	Axon guidance	30/646	128/5712	6.43E-05	0.00163119	SEMA3F/SEMA3B/SEMA3C/SEMA5B/SEMA6A/SEMA4G/NFATC2/LIMK1/EFNB3/SEMA5A/GNAI2/EFNB2/RAC2/PLXNA3/NFATC1/SEMA4F/SEMA7A/SEMA6C/RASA1/ITGB1/ROBO3/PLXNB1/EFNA1/RHOD/ABLIM3/LIMK2/SEMA4B/EPHB4/PLXNB3/L1CAM	30
KEGG	hsa05200	Pathways in cancer	59/646	323/5712	9.52E-05	0.00214743	RALA/DAPK2/CDH1/CTNNA1/LAMA3/TRAF1/LAMC2/TRAF5/MMP2/PDGFB/HIF1A/MMP9/TGFB1/WNT5B/PPARD/VEGFA/FGF1/FN1/SLC2A1/PGF/PLCG1/PAX8/RAC2/EGLN3/CCNA1/COL4A2/LAMC1/ITGAV/FGF5/IGF1R/PML/TP53/SMAD4/ARNT/WNT9A/ITGB1/PRKCA/WNT7A/RUNX1/FGF11/JAK1/WNT4/TGFA/ITGA2/SHH/DAPK3/CTNNB1/CXCL8/PIK3CD/LAMB2/JUN/CSF1R/COL4A1/COL4A5/MMP1/LAMB3/PDGFA/COL4A6/PLCG2	59
KEGG	hsa05412	Arrhythmogenic right ventricular cardiomyopathy (ARVC)	20/646	74/5712	0.00013837	0.00280898	CTNNA1/ACTN1/ITGB5/DSP/ITGB8/ITGB6/ITGB4/DSC2/ITGA7/ITGAV/CACNG8/ITGB1/GJA1/ITGA5/SGCB/ITGA2/CACNB3/CTNNB1/ACTG1/ITGB3	20
KEGG	hsa00511	Other glycan degradation	8/646	17/5712	0.00024517	0.00452457	MAN2B2/AGA/MAN2B1/MANBA/MAN2C1/ENGASE/GLB1/HEXA	8
KEGG	hsa04514	Cell adhesion molecules (CAMs)	28/646	130/5712	0.00050535	0.00854884	CD99/CDH1/CDH3/NEO1/GLG1/CD276/ITGB8/NECTIN1/SDC1/CD58/SDC4/ITGAV/PTPRF/ITGB1/CLDN14/SDC3/NFASC/SDC2/ITGAM/NLGN2/PTPRM/PDCD1LG2/L1CAM/HLA-C/HLA-E/HLA-F/HLA-A/HLA-B	28
KEGG	hsa00531	Glycosaminoglycan degradation	8/646	19/5712	0.00061996	0.00968096	NAGLU/HYAL1/IDUA/GALNS/HGSNAT/GUSB/GLB1/HEXA	8
KEGG	hsa05410	Hypertrophic cardiomyopathy (HCM)	19/646	83/5712	0.00186048	0.026977	ITGB5/TGFB1/ITGB8/ITGB6/PRKAB2/ITGB4/ITGA7/ITGAV/TPM1/CACNG8/ITGB1/TTN/ITGA5/SGCB/ITGA2/TPM4/CACNB3/ACTG1/ITGB3	19
KEGG	hsa05414	Dilated cardiomyopathy	20/646	90/5712	0.00209025	0.02828799	ITGB5/TGFB1/ITGB8/ITGB6/ADCY7/ADCY4/ITGB4/ITGA7/ITGAV/TPM1/CACNG8/ITGB1/TTN/ITGA5/SGCB/ITGA2/TPM4/CACNB3/ACTG1/ITGB3	20
KEGG	hsa04974	Protein digestion and absorption	18/646	80/5712	0.00297038	0.03768667	COL17A1/SLC9A3/COL5A3/SLC15A1/SLC7A8/SLC1A1/COL1A1/SLC36A1/COL5A1/COL4A2/COL6A1/COL6A2/COL3A1/COL18A1/COL4A1/COL4A5/COL4A6/COL5A2	18
KEGG	hsa04380	Osteoclast differentiation	25/646	126/5712	0.00330019	0.0394081	CYBA/FOSL2/NCF4/NFATC2/ACP5/RELB/TGFB1/TYK2/FHL2/NCF2/FOSB/NFATC1/MAPK13/IFNAR2/IFNGR2/SQSTM1/JAK1/OSCAR/JUNB/PIK3CD/JUN/CSF1R/MAPK11/PLCG2/ITGB3	25
KEGG	hsa05100	Bacterial invasion of epithelial cells	16/646	70/5712	0.00421057	0.04748589	VCL/CDH1/CTNNA1/CLTCL1/CTTN/PXN/FN1/SHC2/SHC3/ITGB1/ITGA5/CTNNB1/PIK3CD/CLTB/ACTG1/SRC	16
